# Active Phase of Nickel Electrocatalysts Driving Alkaline Hydrogen Evolution

**DOI:** 10.1002/adma.74591

**Published:** 2026-08-12

**Authors:** Yifeng Wang, Eleanor Ender, Santosh Kumar, Cindy Tseng, Guangmeimei Yang, Boxi Ye, Caiwu Liang, Youli Yu, Norton West, Inderjeet Chauhan, Jun H. Ng, Sid Halder, Sang Gu Ji, Georg Held, Mary P. Ryan, Katie L. Moore, Alex S. Walton, Reshma R. Rao

**Affiliations:** ^1^ Department of Materials Imperial College London London U.K; ^2^ Photon Science Institute The University of Manchester Manchester U.K; ^3^ Department of Materials The University of Manchester Manchester U.K; ^4^ Diamond Light Source Didcot U.K; ^5^ Department of Chemistry Imperial College London London U.K

**Keywords:** electrocatalysts, hydrogen evolution, nickel, *operando* spectroscopy

## Abstract

Nickel‐based cathodes are widely used in alkaline water electrolysis, yet the nature and stability of the active surface under operating conditions remains unclear. In particular, the role of metal/oxo–hydroxo interfacial structures in governing hydrogen evolution activity is not well understood. Here, we employ a multimodal, depth‐sensitive approach combining *operando* Ni L‐edge X‐ray absorption spectroscopy, depth‐sensitive X‐ray absorption measurements in total electron yield and Auger electron yield modes, X‐ray photoelectron spectroscopy, isotopically labeled nano secondary ion mass spectrometry, and online electrochemical mass spectrometry to directly track the evolution of Ni/NiO_x_H_y_ interfaces during the hydrogen evolution reaction. Using well‐defined sputtered Ni thin films as a model system, we show that progressive reduction of near‐surface oxide/hydroxide species is accompanied by a gradual loss of hydrogen evolution activity. Depth‐resolved measurements reveal a predominantly metallic outermost surface under cathodic bias, while NiO_x_H_y_ forms on the surface upon relaxation to open‐circuit conditions. Importantly, mild anodic pre‐conditioning regenerates subsurface NiO_x_H_y_ species, resulting in a sustained increase in hydrogen evolution activity upon subsequent cathodic polarization. These results establish the crucial role of metal/oxo–hydroxo interfaces as active phases for hydrogen evolution and provide a framework for engineering robust, Earth‐abundant HER cathodes capable of operating under dynamic, real‐world electrolysis conditions.

## Graphical abstract

Using depth‐resolved and operando spectroscopic measurements coupled with electrochemical mass spectrometry, the authors uncover the critical role of sub‐surface oxidised species in increasing the activity and durability of Ni‐based cathodes for hydrogen evolution.

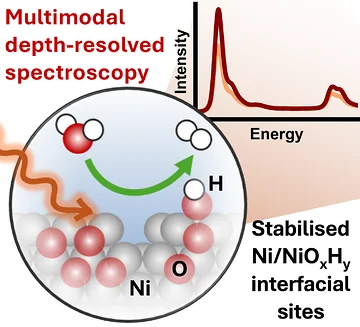

**Abbreviations**
AEY: Auger electron yield
EC–MS: electrochemistry coupled with mass spectroscopy
HER: hydrogen evolution reaction
NEXAFS: near edge X‐ray absorption fine structure
RHE: reversible hydrogen electrode
SEM: scanning electron microscopy
TEM: transmission electron microscopy
TEY: total electron yield
TFY: total fluorescence yield
XAS: X‐ray absorption spectroscopy
XPS: X‐ray photoelectron spectroscopy

## 1 Introduction

Alkaline water electrolysis (AWE) is the most commercially mature technology for large‐scale green hydrogen production, yet the rational design of earth‐abundant electrodes remains constrained by limited understanding of the catalytic interface under operating conditions and the knowledge of the active site responsible for the evolving hydrogen [1, 2, 3, 4, 5, 6, 7]. The hydrogen evolution reaction (HER) has long served as a model electrochemical reaction for studying catalytic structure–activity relationships, with theoretical frameworks linking activity to hydrogen adsorption energetics and establishing the well‐known volcano relationship between catalytic activity and hydrogen binding energy [8, 9]. In alkaline media, HER kinetics are substantially slower than in acidic environments because the reaction requires prior water dissociation (Volmer step) before hydrogen adsorption can occur, complicating this picture significantly [10, 11, 12, 13].

In AWE, nickel‐based cathodes have been widely adopted [2, 14, 15, 16, 17, 18]; however, the precise chemical nature of the active site that controls their activity and stability under variable operating conditions remains elusive [11, 19, 20]. Nickel readily passivates upon air exposure, forming a thin surface layer composed of NiO_x_H_y_ over metallic Ni [21]. Yet under variable load, its surface can evolve dynamically, creating complex, potential‐dependent interfaces involving Ni metal, NiO, Ni(OH)_2_ and potentially NiH_x_ [20, 21, 22, 23]. While the hydrogen evolution reaction itself is intrinsically reductive and bulk thermodynamic predictions indicate metallic Ni as the most stable phase under HER conditions [24], the extent to which these predictions capture the actual interfacial chemistry and the timescale over which NiO_x_H_y_ is reduced under operation remain unclear. This is particularly relevant for device operation, where under shut down conditions, the potential at the cathode can reach up to ∼1 V_RHE_ [25], resulting in oxidation of Ni centres. Ni‐based cathodes also suffer from severe long‐term degradation under operating conditions, which has been often attributed to nickel hydride formation, although the direct *operando* spectroscopic validation of the formation of hydrides remains limited [21, 26]. Consequently, despite its significance as a cathode catalyst for AWE, many uncertainties remain about the evolution of nickel catalysts under operating conditions, and the factors driving its activity and stability [3].

Previous efforts to improve Ni based electrodes have pursued a range of surface modifications and compositional strategies [17, 23, 27, 28, 29, 30, 31, 32, 33], with recent studies focusing on engineering the composition of metallic and oxide species in the sample [20, 22, 31, 34, 35, 36, 37, 38]. Beyond optimizing for activity, the role of surface composition of nickel is very relevant from a device operation standpoint, where under practical operation with fluctuating power supply, sudden potential reversals or transient anodic currents can result in nickel oxidation [25]. In this regard, Gong et al. [20] demonstrated that nanoscale Ni/NiO heterostructures exhibit promising alkaline HER activity, despite both Ni and NiO being intrinsically inactive when isolated, revealing that catalytic enhancement originates from synergistic metal–oxide interfaces rather than from either phase alone. Formation of oxo species upon anodic polarization has also shown to have a beneficial influence on HER kinetics on nickel [39]. Similarly, Subbaraman et al. [11] established a bifunctional mechanism with Ni(OH)_2_ deposited on Pt(111) surface, where Ni(OH)_2_ domains promote water dissociation while adjacent Pt sites facilitate hydrogen adsorption and recombination, dramatically accelerating alkaline HER kinetics. Similar hypothesis has also been proposed recently by Brige et al. [40] with dip‐and‐pull X‐ray photoelectron spectroscopy (XPS) and Finsgar et al. [34] using Time of flight secondary ion mass spectrometry (ToF‐SIMS) and theoretical modeling.

Conflicting reports currently debate whether high HER activity arises from a purely metallic surface, a persistent metastable oxide/hydroxide interface, or transient subsurface species [20, 21, 31, 34, 36, 40, 41]. The role of surface or subsurface oxides has been under debate not solely for the HER reaction [42, 43, 44], but also for CO_2_ reduction [35, 43, 45, 46, 47]. Recent work by Zhou et al. [45] showed that Ni catalysts formed by reduction of different precursors (nickel phosphate, nickel hydroxide, and nickel oxide) can alter the binding energy of oxygen‐containing species by changing the degree of charge polarization on the Ni sites, and thus modify the product selectivity. Likewise, DFT calculations by Dong et al. [42] show that the hydrogen absorption energy on Ni (111) surface could also benefit from a small coverage of oxygen near the surface by tuning the electronic properties of Ni to an optimal level. Interestingly, fully oxidized nickel phases such as NiO and Ni(OH)_2_ are poor HER catalysts when present as stand‐alone materials, owing to their low electronic conductivity and unfavorable hydrogen adsorption energetics, which limit charge transfer and hinder the Volmer step in alkaline media [20, 21]. The dynamic and complex interplay between surface oxidation, subsurface oxides, and metallic Ni availability remains poorly defined, representing a critical gap in understanding the evolution of species *operando* and the design of durable, high‐activity Ni cathodes.

To bridge the gap between empirical observations and mechanistic understanding, we employ a multimodal, depth‐resolved strategy integrating *operando* Ni L‐edge X‐ray near‐edge X‐ray absorption fine structure (NEXAFS), surface‐ and near‐surface‐sensitive total electron yield (TEY), Auger electron yield (AEY), and XPS, together with NanoSIMS using isotopic labelling and online electrochemistry–mass spectrometry (EC–MS). We demonstrate that under HER conditions, the outermost surface layer is mostly metallic Ni, resulting in the formation of a Ni(OH)_2_ layer upon relaxation back to open circuit conditions. Upon anodic oxidation, a thick (oxy)hydroxide layer forms, which persists upon exposure back to HER potentials. The presence and evolution of these oxides are closely linked to the observed HER performance. Progressive reduction of the oxide fraction during operation is accompanied by a gradual loss of catalytic activity, indicating that both surface and near‐surface oxide/hydroxide species are essential to sustain catalytic activity. The Ni/NiO_x_H_y_ interfacial states that persist under HER conditions can be deliberately generated by mild anodic pre‐conditioning, leading to reproducible catalytic enhancement. More broadly, these findings provide a framework for designing robust, earth‐abundant HER cathodes that leverage deliberate metal/oxo–hydroxo interfacial engineering by tuning composition and operating conditions to improve HER performance and durability.

## 2 Results and Discussion

### 2.1 Sample Synthesis and Characterization

Magnetron‐sputtered Ni thin films with nominal thicknesses of 30 nm were prepared on glassy carbon substrates with a 10 nm Au adhesion layer (Figure 1A). As seen in Figure S1 and Figure 1B, profilometry results and the scanning electron microscopy (SEM) images show a well‐defined, relatively flat film with particle sizes of ∼15 nm. To probe the chemical state as a function of sample depth, multimodal *operando* and ex‐situ X‐ray spectroscopy, NanoSIMS and on‐line electrochemical mass spectrometry techniques were used, as shown in Figure 2 and Table S1. Ex‐situ Ni K‐edge EXAFS, with a probing depth spanning the entire film thickness, was first used to determine the bulk chemical state of the sputtered film. As shown in Figure 1C, the absorption spectrum closely resembles that of metallic Ni foil, with only weak features characteristic of NiO and Ni(OH)_2_ around ∼8350 eV, indicating that the film is predominantly metallic. The Fourier transform of the EXAFS signal does not show a characteristic peak at ∼1.7 Å, corresponding to a Ni─O bond [48]. These results thus confirm that the bulk of the sample is metallic in nature. Ni 2p XPS (Figure 1D and Figures S2–S5; Table S2) shows contributions from metallic Ni signal and surface oxo species, suggesting the presence of surface hydroxides/oxides [49, 50]. Considering the probing depth of the Ni 2p XPS is ∼1.6 nm (Figure 2), we can conclude that the thickness of the native surface oxo/hydroxo layer is less than ∼1.6 nm.

**FIGURE 1:**
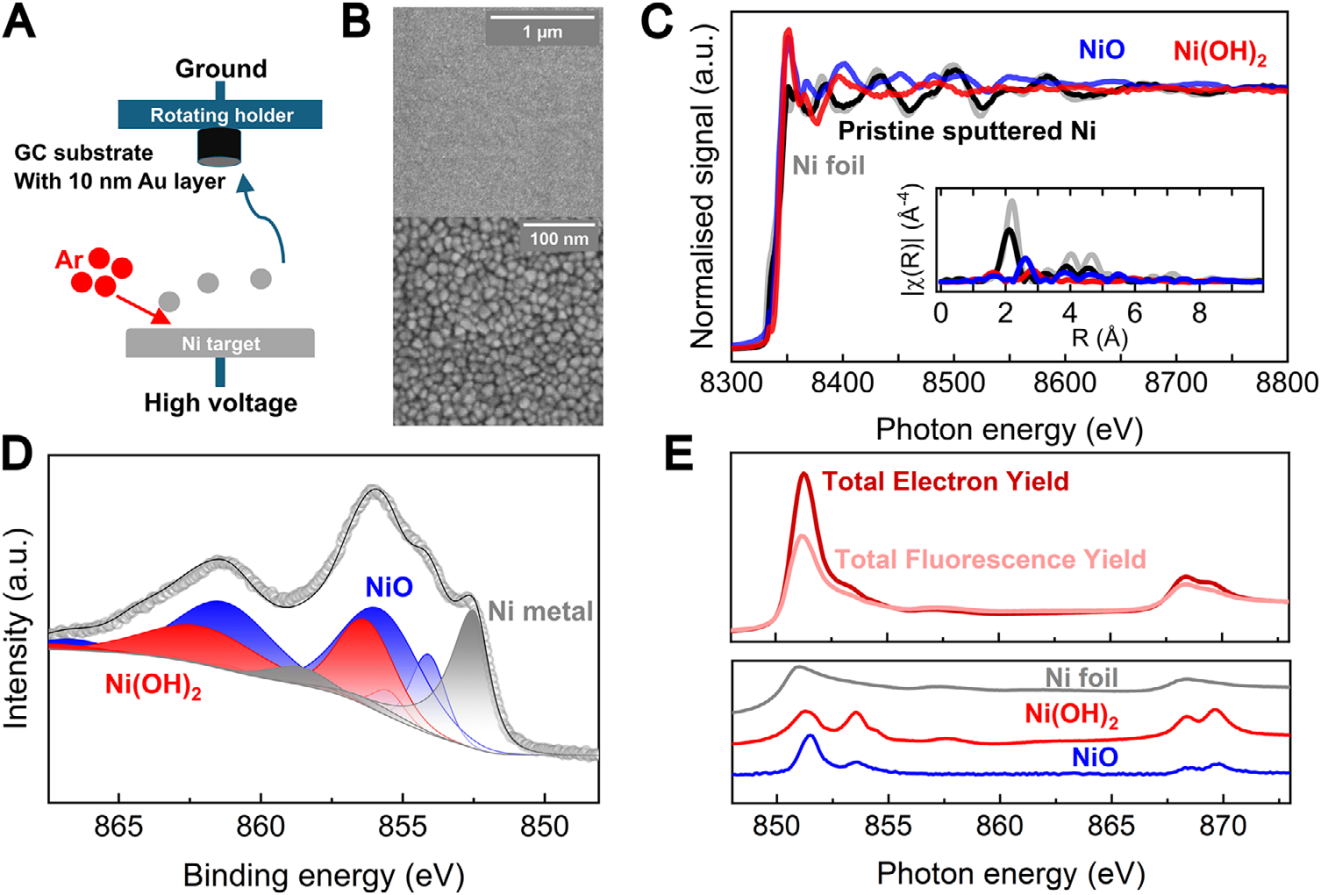
(A) Schematic showing the sputter deposition process used to form planar films of nickel (B) Scanning electron microscope (SEM) images of the nickel deposited on glassy carbon substrate. (C) Ni K‐edge X‐ray absorption near edge structure (XANES) for the as‐deposited sample (black) and reference samples including Ni foil (grey), NiO (blue) and Ni(OH)_2_ (red). The inset shows the corresponding extended X‐ray absorption fine structure (EXAFS); (D) Ni 2p X‐ray photoelectron spectroscopy (XPS) spectrum for the as‐prepared samples measured at 1200.0 eV incident X‐ray (probing depth ∼ 1.6 nm, detailed calculations can be found in Supporting Information); (E) Ni L‐edge near edge X‐ray absorption fine structure (NEXAFS) spectra measured in the total electron yield mode (TEY, dark red) and total fluorescence yield mode (TFY, light red) for the as‐prepared samples, scaled spectra for Ni foil, Ni(OH)_2_ and NiO are also shown for reference (note that the standards spectra here are scaled to a similar intensity for clarity, see Figure S6 for the unscaled spectra). Ni(OH)_2_ and NiO show increase in intensity at ∼851.5, ∼853.5, and ∼854.5 eV compared to metallic Ni foil.

**FIGURE 2:**
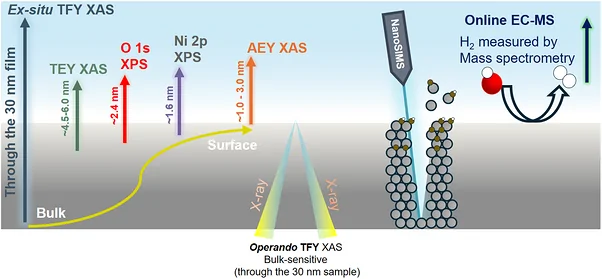
Schematic illustration of the ex‐situ and *operando* characterization techniques employed to probe the nickel–electrolyte interface in this study. The estimated depth resolution associated with each technique is indicated in the figure; details of the corresponding calculations are provided in the Supporting Information.

These findings are corroborated by the contrast between Ni L‐edge TEY and TFY measurements of the pristine 30 nm thin film in Figure 1E, where the shoulder at ∼853.5 eV seen in TEY (probing depth ∼4.5–6.0 nm) is characteristic of oxide/hydroxide species. On the contrary, TFY measurements, which measure the entire thickness of the 30 nm film show a much smaller contribution from this shoulder. Together, these observations confirm that the film consists of a metallic Ni bulk covered by a thin native passive oxide layer composed of NiO and Ni(OH)_2_.

The polarization curve for the 30 nm sputtered Ni film is shown in Figure 3A. As seen in the inset and Figure S7, a redox peak is observed at ∼0.03 V_RHE_, prior to the onset of HER. This redox process has been widely attributed to reduction of NiO/Ni(OH)_2_ species to metallic nickel, suggesting that at least partial reduction of the oxidized species occurs during the evolution of hydrogen [21, 40, 51]. As shown in Figure 3B, as the cathodic current measured during HER can originate from both hydrogen evolution and electrochemical reduction of the native NiO_x_H_y_ passive layer, online electrochemistry–mass spectrometry (EC‐MS) measurements were used to measure the amount of hydrogen gas evolved. Based on calibration of the hydrogen signal (Figure S8), the total charge passed during the 1500 s reduction process of the pristine sample at ‐0.09 V_RHE_ can be further deconvoluted into contributions from HER and oxide reduction, assuming these two processes are the only reactions occurring. Interestingly, the slow decay of current beyond the first few seconds (−0.38 mA cm^−2^ at 500 s to −0.24 mA cm^−2^ at 1500 s) corresponds to a similarly slow decrease in hydrogen production as well as the increase in charge passed for oxide reduction, suggesting progressive deactivation of the catalyst under constant potential conditions is correlated to its reduction. The Faradaic efficiency for hydrogen production is very low during the first few seconds, where a significant fraction of the charge passed results in the reduction of surface oxides. Even after the first 1000 s, faradaic efficiency of hydrogen production is less than 100% (∼75%), with a smaller fraction of the charge contributing to reduction of sub surface oxides. Notably, Mauer et al. [52], also found that holding a nickel catalyst at 100 mA cm^−2^ resulted in an increase in overpotential of ∼400 mV over 15 h of operation.

**FIGURE 3:**
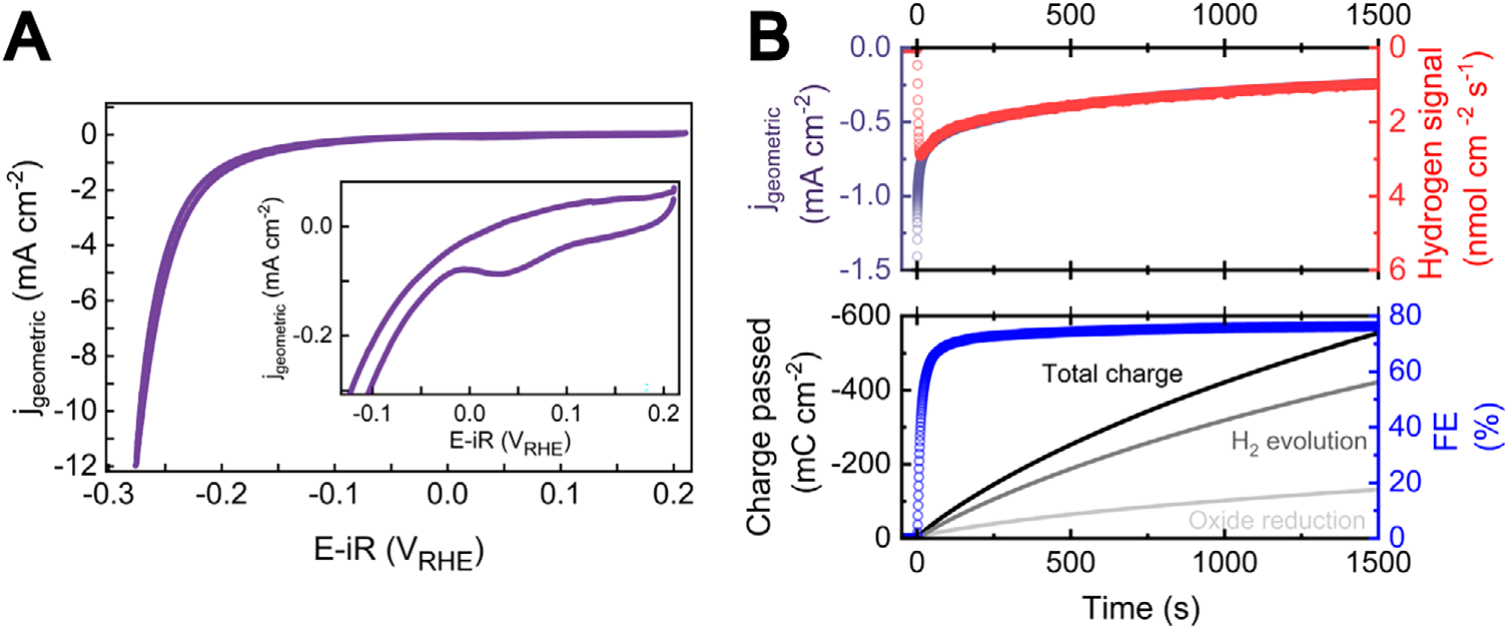
(A) Cyclic voltammogram of the as‐prepared Ni films recorded in Ar‐saturated 0.1 M KOH at a scan rate of 20 mV/s. Lower scan rate CV is available in the Figure S7; (B) Current—time response measured during chronoamperometry (CA) at a constant applied potential of −0.09 V_RHE_ (purple). The hydrogen evolved over the same time interval was quantified by electrochemical mass spectrometry (red points). The lower panel shows the deconvolution of the total charge passed (black) into hydrogen evolution charge (grey) and oxide reduction charge (light grey). Two electrons per hydrogen molecule is assumed. Calibration (Figure S8) and calculations can be found in Supporting Information. A Hg/HgO electrode was used as the reference electrode, and a carbon rod served as the counter electrode, Ar‐purged 0.1 M KOH at room temperature and pressure was used.

Taken together, the online EC–MS data indicate that the early current transient contains a significant non‐HER component associated with reduction of surface oxo/hydroxo species, while the slower decline in hydrogen production reflects gradual loss of catalytic activity under sustained cathodic bias. We therefore propose that progressive reduction of NiO_x_H_y_ species depletes the population of Ni/NiO_x_H_y_ interfacial sites, leading to the observed decrease in hydrogen evolution activity over time.

### 2.2 Evolution of the Nickel Cathode at HER Potentials

To directly probe the chemical evolution of Ni during HER conditions, *operando* Ni L‐edge NEXAFS was collected in TFY mode while applying a series of cathodic potentials. O K‐edge spectra recorded at open‐circuit potential (OCP) are dominated by the water signal, indicating that the TFY probing depth extends through the full film thickness (Figure S9). As shown in Figure 4A, at OCP the spectrum is dominated by metallic Ni, with residual NiO_x_H_y_ features at ∼853.5 eV and ∼869.7 eV. Upon application of increasingly negative potentials from OCP to −0.49 V_RHE_, the Ni L_3_ absorption edge gradually shifts from 851.15 to 851.08 eV (Figure S10) and the NiO_x_H_y_ features (∼853.5 eV) decrease in intensity (Figure S11), demonstrating progressive reduction of the native passive layer during HER. Formation of NiH_x_, although previously reported to be a major deactivation mechanism of Ni cathodes [21, 26], was not observed here. After holding the sample under HER conditions and returning to OCP, NiO_x_H_y_ features partially reappear, together with an irreversible shift of the edge position toward lower energy. This indicates that after exposure to −0.39 V_RHE_, the Ni catalyst remains in an overall more reduced state than in the pristine film.

**FIGURE 4:**
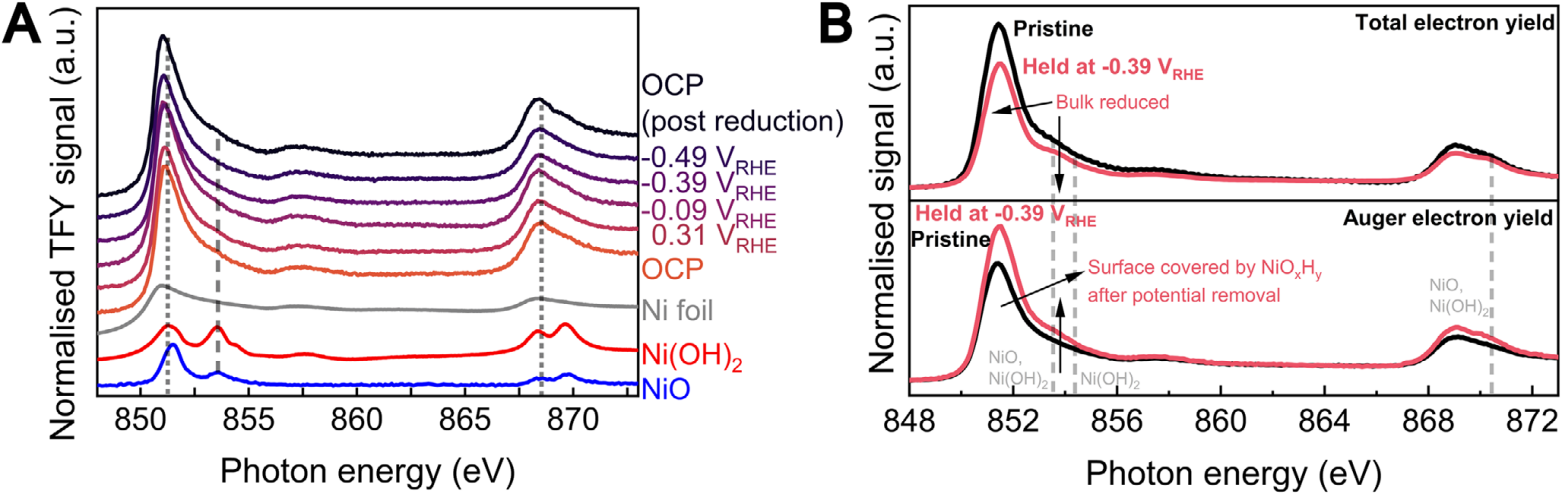
(A) *Operando* Ni L‐edge spectra collected as a function of applied potential from open circuit potential (OCP) to −0.49 V_RHE_. Scaled reference spectra of Ni foil, Ni(OH)_2_, and NiO are included for comparison; (B) Total electron yield (TEY) and Auger electron yield (AEY) Ni L‐edge spectra of the as‐prepared pristine sample (black) and after holding the sample at −0.39 V_RHE_ for 30 min (red).

Since the *operando* NEXAFS measurements presented above were collected in TFY mode and are therefore predominantly bulk sensitive (Figure 2), additional surface‐ and interface‐sensitive techniques are required to disentangle bulk and interfacial contributions to the signal. The in‐situ TFY measurements were thus supplemented with ex‐situ TEY measurements (probing depth ∼4.5–6 nm) and AEY measurements (probing depth ∼1.0–3.0 nm), Figure 4B. TEY measurements show that the sample is more reduced after being held at HER potential of −0.39 V_RHE_, indicative of the presence of more metallic Ni sites being formed upon exposure to HER potentials, consistent with the *operando* TFY data in Figure 4A. On the contrary, the more surface sensitive AEY measurements indicate the top few layers of the sample are more oxidized following exposure of the film to HER potentials. The subsequent appearance of Ni(OH)_2_‐like species at the surface therefore most likely arises during relaxation from reductive HER potentials back to OCP and/or upon brief air exposure between measurements, considering Ni metal has been suggested to have a strong affinity toward hydroxylation, readily forming Ni(OH)_2_ as shown in previous reports [21, 53] and our recent work [54] utilizing near‐ambient pressure XPS and isotopic labelling NanoSIMS. Collectively, these results demonstrate that the catalyst exists in a highly dynamic chemical state, characterized by continuous evolution of a mixed Ni/NiO_x_H_y_ interfaces in response to changes in electrochemical potential.

### 2.3 Role of Oxidized Ni Species in Modulating HER Activity

To assess the role of Ni/NiO_x_H_y_ interfaces on HER kinetics, the Ni thin films were subjected to a mild anodic pre‐conditioning step to introduce higher degrees of oxidation on the pre‐reduced Ni electrodes. As shown in Figure 5A, during the initial 30 min of reductive HER at ‐0.39 V_RHE_, the current density decreases substantially from ∼‐10 to ∼‐6 mA cm^−2^, consistent with the progressive loss of the native Ni/NiO_x_H_y_ interfacial contribution discussed above. Upon application of the anodic pre‐conditioning step (1.31 V_RHE_ here), a cathodic current under HER of ∼‐10 mA/cm^2^ was regained with oxygen uptake confirmed by the NanoSIMS which will be discussed later (Figure S12). Notably, repeated cycles of anodic oxidation result in continual improvement in current (Figures S13 and S14) which could last more than 8 h, suggesting short, minutes of mild oxidation during hours of continuous operation as a viable route to activate Ni cathodes. These results suggest the important role played by surface/sub‐surface oxo species in modulating the HER kinetics.

**FIGURE 5:**
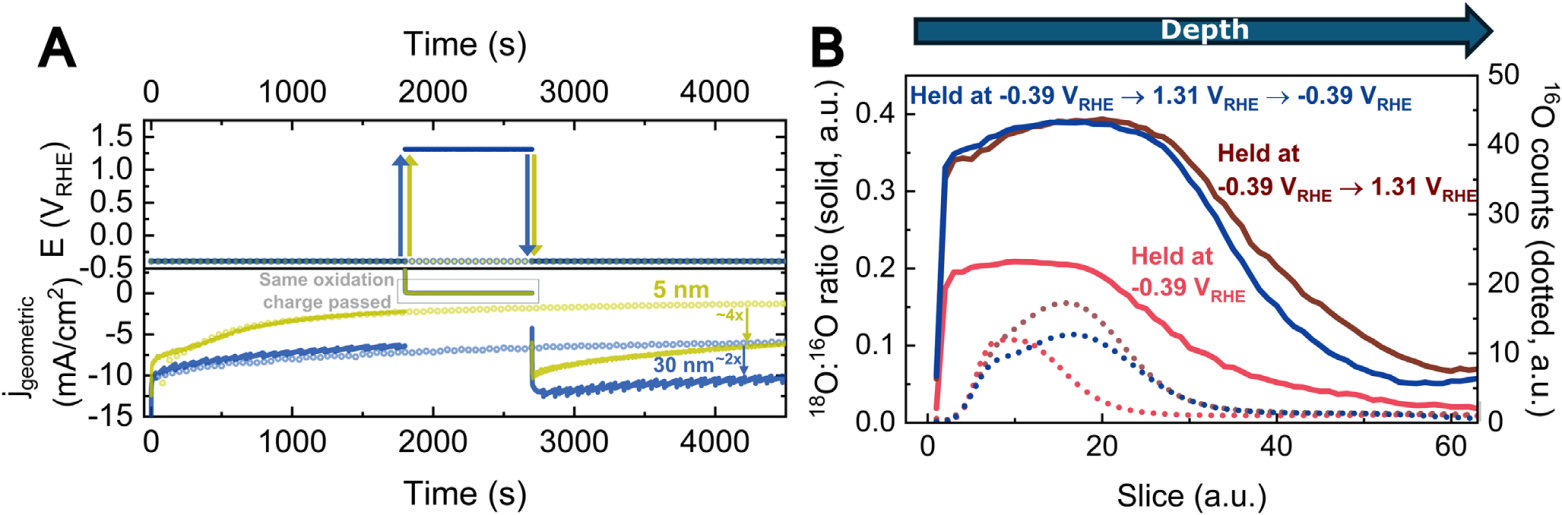
(A) Chronoamperometric response of 30 nm (blue) and 5 nm (green) Ni films during a potential hold at −0.39 V_RHE_ for 30 min, followed by anodic oxidation at 1.31 V_RHE_ for 15 min and subsequent reduction at −0.39 V_RHE_ for 30 min. Zoomed‐in plot of the oxidation charge part is provided in Figure S17; (B) NanoSIMS depth profiles of the 30 nm Ni film after (i) holding at −0.39 V_RHE_ (pink), (ii) holding at −0.39 V_RHE_ followed by oxidation at 1.31 V_RHE_ (brown), and (iii) holding at −0.39 V_RHE_ followed by oxidation at 1.31 V_RHE_ and reduction at −0.39 V_RHE_ (blue). The data show the enrichment of the ^1^ ^8^O signal relative to ^1^ ^6^O as a function of depth into the sample (solid lines) and the ^16^O counts (dotted lines). All electrochemical measurements were performed in Ar‐saturated 0.1 M KOH using a Hg/HgO reference electrode and a carbon rod counter electrode. NanoSIMS measurements were conducted in ^2^H_2_^1^ ^8^O‐containing electrolyte. The raw NanoSIMS counts are available in Figure S12.

Therefore, we next compare the electrochemical response of a 30 nm film with a significantly thinner 5 nm sample, where a larger fraction of the sample would have oxo species. This is confirmed by the TEY and TFY NEXAFS at Ni L‐edge, which shows that the prominent shoulder at ∼853.5 eV (corresponding to NiO_x_H_y_) for the TEY measurements, which is diminished for the bulk sensitive TFY measurements. However, for the thinner 5 nm sample, a similar intensity of the NiO_x_H_y_ features is observed, further confirming that a larger fraction of the sample has NiO_x_H_y_ (Figure S15). The 5 nm film exhibits a lower absolute current density than the 30 nm film, in agreement with its smaller electrochemically active surface area (Figure S16). Upon application of the anodic pre‐conditioning step (1.31 V_RHE_ here), however, the oxidation charges, obtained by integrating the current–time response, are similar for the 5 nm and 30 nm films (Figure 5A and Figure S17). This suggests the degree of oxidation is independent of the film thickness. This interpretation is further supported by the comparable charges associated with the Ni(OH)_2_ + OH^−^ → NiOOH + H_2_O + e^−^ redox wave observed for both films (Figure S18), suggesting that only a limited thickness of NiO_x_H_y_ can be generated within the accessible timescale, irrespective of the total film thickness. When the electrodes are subsequently returned to reductive HER potentials, both samples exhibit an increase in current density relative to continuous HER operation without pre‐conditioning. Notably, the 5 nm and 30 nm samples exhibit two key differences—(1) the rate of HER activity loss is much more pronounced for the thinner sample, both pre‐ and post‐ the application of anodic bias (Figure 5A), and (2) the degree of increase in HER activity is significantly larger for the thinner sample. These differences suggest that the anodically formed NiO_x_H_y_ species are more readily reduced in the thinner film, consistent with a larger fraction of the film participating in reversible interfacial redox processes. This results in a more significant loss of HER activity as a function of time. Furthermore, the stronger influence of anodic oxidation on the HER kinetics of the 5 nm film compared to the 30 nm film also suggests that the larger fraction of film being oxidized for the 5 nm sample is beneficial to the HER kinetics. The thickness dependent electrochemical results therefore suggest the critical role being played by oxides, formed at the surface and sub‐surface, on the activity and stability of nickel cathodes.

To further probe the role of the applied anodic potential, the 30 nm films were pre‐conditioned at different anodic biases (Figure S19). Pre‐conditioning at 0.91 and 1.31 V_RHE_, corresponding primarily to OH^−^ uptake and thickening of the NiO_x_H_y_ layer, result in enhanced HER currents upon returning to −0.39 V_RHE_. In contrast, pre‐conditioning at 1.71 V_RHE_, which lies well within the OER regime and involves further oxidation steps (Ni(OH)_2_ + OH^−^ → NiOOH + H_2_O + e^−^ and NiOOH + OH^−^ → NiOO + H_2_O + e^−^ [21, 55, 56].), leads to a lessened enhancement, indicating the over‐oxidation [21]. However, there is still an enhancement from the sample without any pre‐conditioning [21]. The results indicate an optimal degree of oxidation could lead to the highest activity. It is worth noting that as the *operando* NEXAFS data is measured in fluorescence mode, which is inherently a bulk technique, quantification of the oxidation state of the active site is challenging. Yet qualitative agreement between the NiO_x_H_y_ formed and HER activity can be shown by correlating the HER current density to the charge passed during anodic pre‐conditioning (Figure S19). To directly track oxygen incorporation, NanoSIMS measurements were performed on a (1) pristine sample, (2) sample exposed to −0.39 V_RHE_, (3) sample exposed to −0.39 V_RHE_ followed by anodic oxidation at 1.31 V_RHE_, and (4) a sample held at −0.39 V_RHE_, followed by anodic oxidation at 1.31 V_RHE_ then −0.39 V_RHE_. Measurements were carried out in ^18^O labelled 0.1 M KOH to disentangle signals from oxidation from air. No ^18^O signal was found in the pristine sample, suggesting any oxide species detected on this sample were from ambient oxidation in air (Figure S12). Following electrochemical measurements in ^18^O labelled 0.1 M KOH, enhanced ^18^O signals were observed, attributed to reactions of the sample with the electrolyte. As shown in Figure 5B the sample exposed to HER at −0.39 V_RHE_ for 30 min followed by application of anodic potential at 1.31 V_RHE_ for 15 min exhibits a substantially enhanced ^18^O signal near the surface compared to pristine and reduced samples, indicating significant oxygen uptake and NiO_x_H_y_ formation. Following the second reduction step, the ^1^ ^8^O signal decreases slightly but remains appreciably higher than in the pristine or initially held at −0.39 V_RHE_ sample, demonstrating partial retention of the newly formed Ni/NiO_x_H_y_ interfacial structure. Assuming that the depth at which ^16^O signal appears is the accessible depth of the native oxide, the samples post oxidation at 1.31 V_RHE_ have significantly larger enhancement of the ^18^O:^16^O signal, deeper within the sub‐surface. Taken together with the thickness‐dependent electrochemical trends, these results indicate that anodic pre‐conditioning drives significant oxygen incorporation beyond the native passive layer and into the sublayer.

To probe the dynamic evolution of the Ni/NiO_x_H_y_ interface under operating conditions, *operando* Ni L‐edge XAS was performed on the 30 nm film during the initial HER measurements, followed by anodic oxidation and subsequent application of HER‐relevant potentials. As shown in Figure 6A and the corresponding *operando* O K‐edge in Figures S20 and S21 and ex‐situ XPS measurements (Figure S22, 1.31 V_RHE_), upon switching to 1.71 V_RHE_, where the highest degree of anodic oxidation is expected, as discussed above, the spectra show a pronounced shoulder at approximately 854 eV. This feature has been attributed to the formation of highly oxidized Ni species (Ni^3/4+^‐like character [57, 58]) under OER‐relevant conditions [56, 59, 60]. Consistent with this assignment, the absorption edge shifts toward higher photon energy, indicating an overall increase in the oxidation state of the catalyst during the anodic step. Notably, when the potential is subsequently returned to reductive HER conditions (‐0.39 V_RHE_), residual NiO and Ni(OH)_2_ spectral contributions remain clearly detectable, demonstrating that the anodically generated Ni/NiO_x_H_y_ interfacial structure is not fully eliminated upon reduction. These retained oxidized features persist even after 5 h of continuous HER operation, accompanied by a sustained enhancement in catalytic current density (Figure 6B). This observation confirms that the in‐situ generated Ni/NiO_x_H_y_ interface is partially retained under the time periods measured here, contributing to sustain HER activity.

**FIGURE 6:**
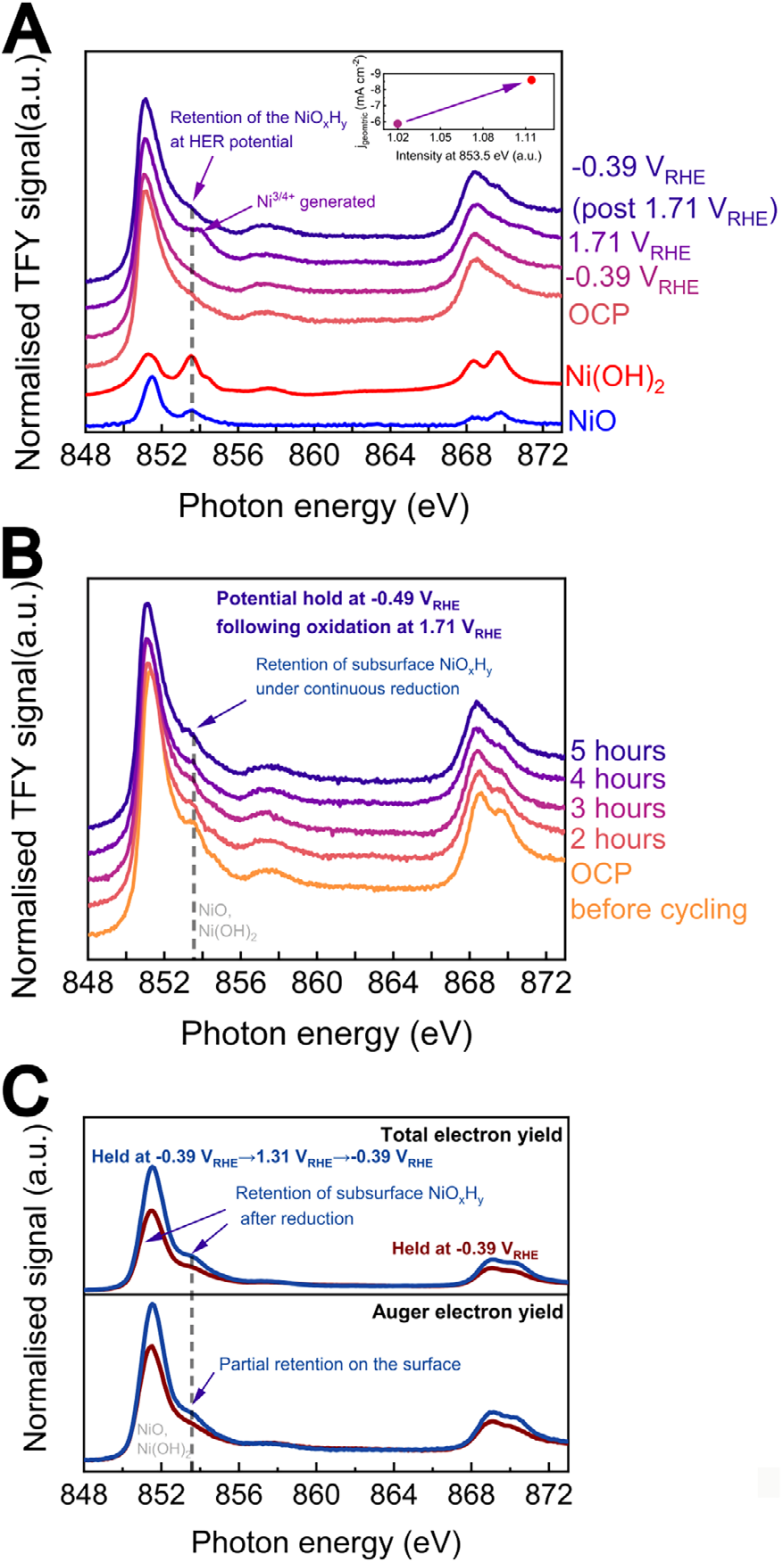
(A) *Operando* Ni L‐edge spectra acquired as a function of applied potential. A reductive potential of −0.39 V_RHE_ was first applied, followed by anodic oxidation at 1.71 V_RHE_ and subsequent reduction back to −0.39 V_RHE_. Scaled reference spectra of Ni(OH)_2_ and NiO are included for comparison. The inset shows the intensity of the NiO_x_H_y_ feature at 853.5 eV, as well as the current density of the nonpre‐conditioned sample and the sample conditioned at 1.71 V_RHE_ under the subsequent HER step as shown in Figure S19A. (B) *Operando* Ni L‐edge spectra collected as a function of time, with the electrode held at −0.49 V_RHE_ following being subject to anodic oxidation at 1.71 V_RHE_. (C) Total electron yield (TEY) and Auger electron yield (AEY) Ni L‐edge spectra of a sample exposed only to reducing potentials of −0.39 V_RHE_ (brown), and of a sample exposed to reducing potentials of −0.39 V_RHE_ following prior reducing potentials of −0.39 V_RHE_ and anodic oxidation at 1.31 V_RHE_ (blue, which yields the maximum activity).

Ex‐situ Ni L‐edge NEXAFS spectra collected in TEY (4.5–6.0 nm probing depth) and AEY (1.0–3.0 nm probing depth) modes provide further information about the location of oxo species within the samples (Figure 6C). The sample held at −0.39 V_RHE_ following anodic pretreatment at 1.31 V_RHE_ exhibits a more oxidized character relative to the pristine and reduced electrodes, measured in both the TEY and AEY modes. In the TEY mode, with measuring depth of 4.5–6.0 nm, a significant increase in NiO_x_H_y_ species (shoulder at ∼853.5 eV) was detected in the samples held at −0.39 V_RHE_ following anodic pretreatment at 1.31 V_RHE_ compared to the samples that were not pre‐oxidized. Similar increase in NiO_x_H_y_ species was observed for AEY measurements with a probing depth of 1.0 – 3.0 nm. However, the increase in NiO_x_H_y_ species for the samples with pre‐oxidation compared to those that were not pre‐oxidized was not as pronounced as that measured in the TEY mode. These results thus suggest that while anodic pre‐oxidation increases the NiO_x_H_y_ at the surface and sub‐surface of the sample, the increase in sub surface NiO_x_H_y_ species is much more prominent. This is further supported by ex‐situ depth‐profiling XPS (Figure S5). Together, these results establish that anodic pre‐conditioning produces a metastable, yet persistent Ni/NiO_x_H_y_ interfacial phase that is at least partially retained under cathodic operation and constitutes the chemically distinct active state responsible for HER activation (Scheme 1).

**SCHEME 1:**
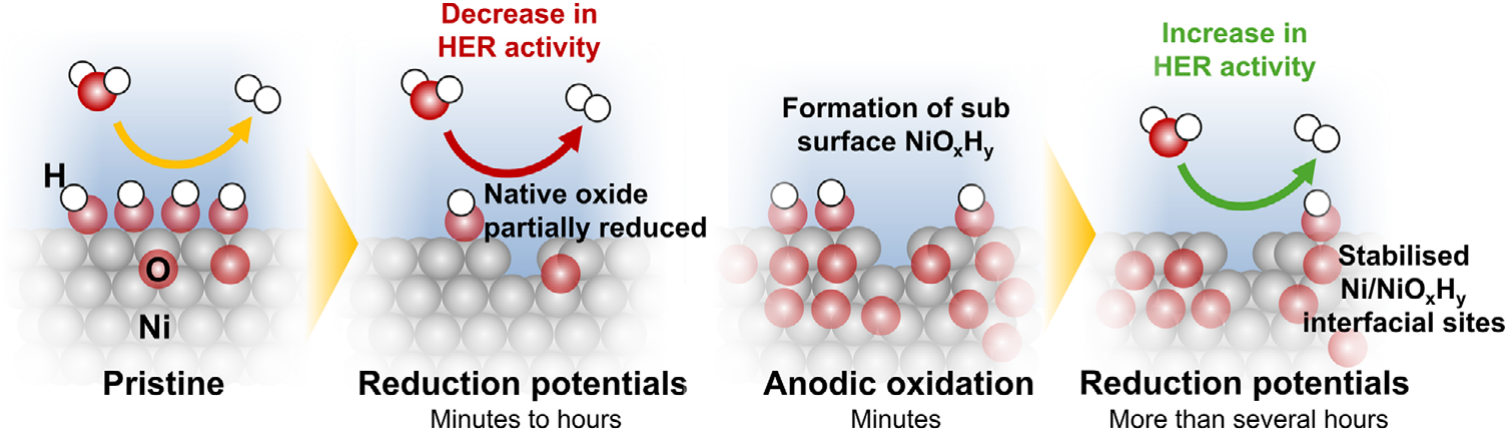
Proposed active phases of nickel during the hydrogen evolution reaction (HER). The as‐prepared samples possess a native oxide/hydroxide layer, which is reduced upon application of HER potentials, accompanied by a decrease in HER activity. Subsequent anodic oxidation generates a thicker oxide layer, which leads to enhanced HER activity upon reduction. Under HER conditions, the surface is proposed to be predominantly metallic, with subsurface NiO_x_H_y_ species contributing to the increased catalytic activity. Grey: Ni, red: O, white: H, atoms are not to scale.

In summary, in this work, we identify that Ni/NiO_x_H_y_ interfaces are the dominant active phase for HER at nickel cathodes, with a metallic surface layer and a more oxidized sub‐surface. NiO and Ni(OH)_2_ are generally regarded as poor HER catalysts when present as standalone bulk phases [11, 20, 21], and their persistence here does not imply that the oxide itself constitutes the active surface. Several possible reasons can explain the increase in activity of the Ni/NiO_x_H_y_ interface. Firstly, in alkaline media, the HER rate is often limited by the Volmer step involving H_2_O dissociation, and interfacial Ni–OH motifs have been proposed to lower the kinetic barrier for this process [61, 62, 63]. It is possible that the metal/oxo heterostructure provides optimized sites for water activation and *H binding. Such a mechanism for activity enhancement could explain why an optimum coverage of metal and oxygenated species is required—a low coverage of either species would result in a loss of the bifunctionality and a large coverage of oxo species would hamper the conductivity. Notably, such bifunctional mechanisms have been widely proposed for Ni(OH)_2_ deposited on Pt [11], as well as other metals such as NiMo [27], MOFs [64] and transition metal phosphides [65]. Secondly, it is possible that the active site remains fundamentally metallic, with the electrode oxidation primarily restructuring the surface into a higher density of undercoordinated Ni sites that act as more efficient single sites for HER. For several catalysts, including the state‐of‐the‐art non‐noble metal based MoS_2_, undercoordinated sites have been reported to be highly active [5, 66]. Notably, we do not observe a change in electrochemically active surface area (Figure S23) and morphology (Figure S25) during anodic oxidation, which suggests that sub‐surface oxo species do not significantly alter the active surface area or the surface morphology while the kinetics sees a significant improvement (Figure S24). The HER activity can be reproducibly restored (Figure S14A) through mild anodic pre‐conditioning. Such reversible recovery is also consistent with reformation of surface/subsurface oxide–hydroxide species than with irreversible structural degradation or bulk hydrogen uptake. Thirdly, another factor that cannot be fully excluded is the involvement of transient surface and subsurface hydrogen in the decrease in HER kinetics, with mild oxidation resulting in partial removal of accumulated NiH_x_ species and thereby restoring metallic surface availability [21]. Finally, incorporation of subsurface oxygen during conditioning could impose lattice strain (as previously observed from NiP_2_ [67], and PtNi [32]) or electronic perturbations on the near‐surface Ni, subtly tuning hydrogen and oxygen binding energetics and promoting HER activity without requiring a fully oxidic surface, instead through controlling the extent of polarization of charges on the nearby Ni sites [45]. Due to the dynamic nature of the catalyst, direct observation of the atomic‐scale active sites during operation requires further investigation and relies on spatially resolved techniques such as *operando* TEY and TFY measurements as well as *operando* diffraction or TEM to determine structural changes at the interface. It is also worth mentioning that, as this study aims to provide a model system and relies on thin film electrodes, the effects of high current density and long operational hours relevant to device operation has not been tested. As the activity enhancement in this study has been shown to encouragingly last for hours with just minutes of pre‐conditioning, testing the protocol at industry‐relevant conditions, using devices with real‐life catalysts such as Ni and its alloys [27, 68, 69, 70], or possibly other emerging materials such as MOF [64] or molybdenum‐based catalysts [5, 68, 71] would be an important future study.

Taken together, these additional pathways highlight that a combination of electronic, structural, and chemical effects arising from the distribution of oxyphilic species in the surface and sub‐surface of the electrode would strongly dictate the performance of Ni catalysts for HER, and controlling the Ni/NiO_x_H_y_ is crucial for engineering activity and durable catalysts as well as optimizing the operational protocol of existing Ni‐based electrolyzers. Considering Ni‐based catalysts are exposed to positive potentials during shut down conditions, due to reverse currents, the role of oxidized species becomes even more crucial for controlling device performance by altering operating conditions. Controlling the degree of oxidation during such intermittent operation could not only prevent degradation of the cathode but potentially regain and improve HER kinetics under operation.

## 3 Conclusions

In this work, we elucidate the dynamic evolution of Ni cathodes under alkaline HER conditions, with particular emphasis on the role of native and electrochemically generated oxide/hydroxide species at the catalyst interface. By combining *operando* Ni L‐edge XAS, depth‐sensitive TEY/AEY/XPS spectroscopy, online EC–MS, and isotopic NanoSIMS tracing, we directly decouple the contributions of bulk metallic Ni from those of surface and subsurface NiO_x_H_y_ species during electrochemical operation. We show that the native passive NiO_x_H_y_ layer undergoes progressive reduction under cathodic HER potentials. However, subsurface NiO_x_H_y_ species partially persist even after extended operation, while the outermost surface becomes predominantly metallic Ni. Importantly, controlled mild anodic conditioning regenerates surface and subsurface oxides/hydroxides, leading to recovery of HER activity. Although the surface exhibits largely metallic character under steady state HER conditions, the presence of subsurface oxides is hypothesized to play a critical role in sustaining catalytic activity on Ni cathodes. More broadly, this study provides a framework for understanding how transient anodic potentials, such as those arising from fluctuating renewable power inputs or reverse currents in electrolyzers, modify surface chemistry and thereby modulate HER kinetics. These insights extend beyond Ni, highlighting that metastable metal/oxo–hydroxo interfaces may represent a general design principle for robust HER electrocatalysts operating under dynamic conditions. Ultimately, engineering and stabilizing such redox‐active interfacial states offers a promising pathway toward durable, earth‐abundant cathodes for next‐generation alkaline water electrolysis.

## 4 Methods

### 4.1 Sample Synthesis and Characterization

Sputter deposition was performed using an AJA Orion‐8 system. A Ni target (AJA) was pre‐cleaned by Ar plasma sputtering (30 mTorr, 5 min) to remove the native passive layer. The sputtering power was adjusted and calibrated using a quartz crystal microbalance (QCM) to obtain a Ni deposition rate of approximately 0.20 Å s^−1^. Polished glassy carbon substrates (HTW, Germany) were transferred to the sputtering chamber and cleaned under the same conditions to remove surface contaminants. A 10 nm Au adhesion layer was first deposited by DC sputtering at ∼0.50 Å s^−1^, followed by deposition of a 30 nm Ni film. Scanning electron microscopy (SEM) was performed using a Zeiss Sigma 300 microscope. Profilometry was done on KLA Alpha‐Step D‐600 stylus profiler.

### 4.2 Electrochemical Measurements

Electrochemical measurements were conducted using a BioLogic SP‐150e potentiostat. Ar‐saturated 0.1 M KOH (99.995%, Sigma–Aldrich) was used as the electrolyte with a Hg/HgO reference electrode (ALS) filled with the same 0.1 M KOH, and a carbon rod counter electrode (Redox.me) was used. An acid‐washed Teflon cell (Redox.me) was used. 1600 rpm rotation rate was used on the PEEK rotator (Pine research). *Operando* measurements were done in a flow cell designed by the beamline, consisting of a PEEK cell body, a micro‐Ag/AgCl micro reference electrode and a Pt rod as counter electrode [72]. Samples were deposited on a SiN_x_ window coated with 10 nm Ti and 10 nm Au. All measurements were done at room temperature and pressure. Online electrochemistry–mass spectrometry (EC‐MS; SpectroInlets) was calibrated against a Pt electrode for Faradaic efficiency determination (see Supporting Information).

### 4.3 *Operando* and Ex‐Situ Spectroscopy

Near edge X‐ray absorption fine structure (NEXAFS) was carried out at the B07 beamline at Diamond Light Source using total fluorescence yield (TFY) for *operando* measurements and reference standards. Total electron yield (TEY), Auger electron yield (AEY), and X‐ray photoelectron spectroscopy (XPS, 1200.0 eV photon energy) were used for ex‐situ characterization [72]. The beam size of the X‐ray is about 50 µm × 50 µm which covers a reasonable amount of sample. Thermo Fisher K‐Alpha+ was used for lab‐based XPS measurements. XPS data was fitted with the CasaXPS software. Extended X‐ray Absorption Fine Structure (EXAFS) and X‐ray absorption near edge structure (XANES) were carried out at the B18 beamline in fluorescence mode.

Isotopic labelling experiments for NanoSIMS analysis were conducted in 0.1 M KOH made up with a 1:1 mix of 97% enriched ^18^O water (CK Isotopes Limited) and 99.9% enriched deuterium oxide (Sigma‐Aldrich). Data was collected on NanoSIMS 50L (CAMECA, France) with a 16 kV Cs^+^ primary ion beam which was scanned over the sample surface to generate negative ions (^1^H^−^, ^2^H^−^, ^12^C^−^, ^12^C^1^H^−^, ^16^O^−^, ^16^O^1^H^−^, and ^18^O^−^) along with the secondary electron image. Depth profiles were acquired by sequentially imaging 200 planes over a 10 µm x 10 µm raster at 256 × 256 pixels using a 0.9 pA beam current and a dwell time of 1000 µs per pixel. To establish a depth profile reconstruction, secondary ion signals were extracted as average counts per plane from the central region of each image to avoid edge effects with no drift correction applied. The D1 aperture was set to 3 (200 µm), and the entrance and aperture slits were set to positions 3 (20 µm) and 2 (200 µm), respectively. The electron multiplier detector voltages were calibrated to achieve peak signals at 210 mV and avoid ageing effects. Image and chemical depth profiles were acquired from a non‐implanted surface starting from the passive (native) oxide layer. Data analysis were performed on ImageJ with the OpenMIMS plugin (Harvard, USA) and L'image (L. R. Nittler, Carnegie Institution of Washington).

## Conflicts of Interest

The authors declare no conflicts of interest.

## Supporting information




**Supporting File**: adma74591‐sup‐0001‐SuppMat.pdf.

## Data Availability

The data that support the findings of this study are available from the corresponding author upon reasonable request.
