# Supplementary material for: Active Phase of Nickel Electrocatalysts Driving Alkaline Hydrogen Evolution
Source: Adv Mater. 2026 Aug 12;38(57):e74591. doi: 10.1002/adma.74591 (PMC13660827; doi:10.1002/adma.74591)
Supplement: Supplementary file 1 [file ADMA-38-e74591-s001.pdf]

Supporting Information

Active Phase of Nickel Electrocatalysts driving Alkaline Hydrogen Evolution

Yifeng Wang,[1] Eleanor Ender,[2,3] Santosh Kumar,[4] Cindy Tseng,[1] Guangmeimei Yang,[5] Boxi Ye,[1] Caiwu Liang, [1] Youli Yu,[1] Norton West,[2,3] Inderjeet Chauhan,[1] Jun H. Ng,[1] Sid Halder,[1] Sang Gu Ji, [1] Georg Held,[4] Mary P. Ryan, [1] Katie L. Moore,[3] Alex S. Walton,[2] Reshma R. Rao[1]*

[1] *Department of Materials, Imperial College London, Exhibition Road, London SW7 2AZ, U.K.*

[2] *Photon Science Institute, The University of Manchester, Oxford Road, Manchester, M13 9PY, U.K.*

[3] *Department of Materials, The University of Manchester, Oxford Road, Manchester, M13 9PL, U.K.*

[4] *Diamond Light Source, Didcot OX11 0DE, U.K*

[5] *Department of Chemistry, Imperial College London, Exhibition Road, London SW7 2AZ, U.K.*

1. Estimation of probing depths of the X-ray based techniques used

1.1. Fluorescence based techniques

Ni K-edge extended X-ray absorption fine structure (EXAFS) was carried out on the B18 beamline at Diamond Light Source using fluorescence mode. Due to the high energy (~8360 eV), it can be deduced that the beam has gone through the 30 nm thick sample.[1]

The *operando* measurement for Ni L-edge and O K-edge were measured at the B07 beamline at Diamond Light Source. The cell configuration[2] determines that incident X-ray has to come in from the back of the sample and the outbound X-ray has to go through the whole of the sample again. Therefore, this technique captures both the bulk and the surface (which is in contact with the electrolyte) information. Operando measurements were conducted in the TFY mode

.

1.2. Electron-based techniques

Electron-based techniques such as total electron yield (TEY), auger electron yield (AEY) and X-ray photoelectron spectroscopy (XPS) were also utilised for ex-situ, depth-sensitive measurements.

For XPS, the angle was at 54.7° and photon energy of the beam was fixed at 1200.0 eV to maximise surface sensitivity, hence:

$$h\nu = 1200.0\ eV$$

For Ni 2p XPS, assuming the Binding energy (BE) ranges from 850 to 870 eV and the work function of the instrument = 4.2 eV [2], thus:

$$E_k = h\nu - BE - \phi = 345.8 \sim 365.8\ eV$$

As the surface of the Ni thin film is covered with NiO_xH_y passive layer as shown in **Figure 1D**, for simplicity the surface can be approximated to be NiO. The inelastic mean free path can be calculated using the NIST standard reference database[3] to be:

$$\lambda \approx 0.9\ nm$$

In this case 95% of the signal can be calculated from a depth of d:

$$d \approx 3\lambda \times \cos(54.7°) \approx 1.6\ nm$$

The same calculation can be done for O 1s XPS (BE = 528 ~ 536 eV):

$$E_k = h\nu - BE - \phi = 659.8 \sim 667.8\ eV$$

$$\lambda \approx 1.4\ nm$$

$$d \approx 3\lambda \times \cos(54.7°) \approx 2.4\ nm$$

Similarly, for lab-based Al K-alpha XPS with 1486.6 eV photon energy and ϕ ~ 4 eV, the probing depth can be calculated to be $d_{Ni\ 2p}$ ≈ 2.31 nm and $d_{O\ 1s}$ ≈ 3.12 nm.

For Ni L-edge XAS, Ni_{2p} to Ni_{3d} transition was probed. TEY measures all emitted electrons (photoelectrons + auger + secondary cascade), whereas AEY measures specifically the auger electrons from Ni LMM. [4]

The effective probing depth in electron-yield XAS is governed by the attenuation length of emitted electrons, such that the detected signal decays exponentially with depth $I(z) \propto \exp(-z/\Lambda)$. The corresponding 95% information depth is approximated as $d_{95} \approx 3\Lambda$. For TEY, dominated by low-energy secondary electrons, $\Lambda \approx 1.5$–2.0 nm, giving $d_{95} \approx 4.5$–6.0 nm. For AEY, based on Auger electrons with shorter effective attenuation lengths ($\Lambda \approx 0.5$–1 nm), the probing depth is limited to $d_{95} \approx 1$–3nm.

Table S1 is a summary of the X-ray based techniques used:

Table S1 Summary of probing depth of spectroscopic techniques used

**Mode**	**Dominant particles**	**Estimated Probing depth**
TFY	Photons	Through film
TEY	Photoelectrons + Auger electrons + secondary cascade	~ 4.5 - 6.0 nm (Ni L-edge)
AEY	Auger electrons	~ 1.0 - 3.0 nm (Ni L-edge)
XPS @ 1200.0 eV photon energy	Photoelectrons	~ 1.6 nm (Ni 2p)<br>~ 2.4 nm (O 1s)
XPS @ 1486.6 eV photon energy	Photoelectrons	~ 2.3 nm (Ni 2p)<br>~ 3.1 nm (O 1s)

The *ex-situ* Ni L-edge AEY XAS signal **(~1.0 – 3.0 nm probing depth)** reports on the outermost surface layer directly in contact with the electrolyte: this is the region most relevant to catalytic interface. The *ex-situ* Ni 2p XPS signal **(~1.6 nm probing depth)** reports on the outermost surface layer directly in contact with the electrolyte: this is the region most relevant to catalytic interface, although slightly deeper than the AEY. The *ex-situ* O 1s XPS signal **(~2.4 nm probing depth)**, similar to the above, the slightly deeper. The *ex-situ* Ni L-edge TEY XAS signal **(~4.5 – 6.0 nm)** captures the near-surface region encompassing the transition from the metallic subsurface to the oxidised surface. The *operando* and ex-*situ* Ni L-edge TFY XAS signal (**through film**) provides a bulk-averaged reference against which surface-specific changes can be distinguished.

2. Profilometry

The flatness and thickness of the samples are assessed with profilometry.

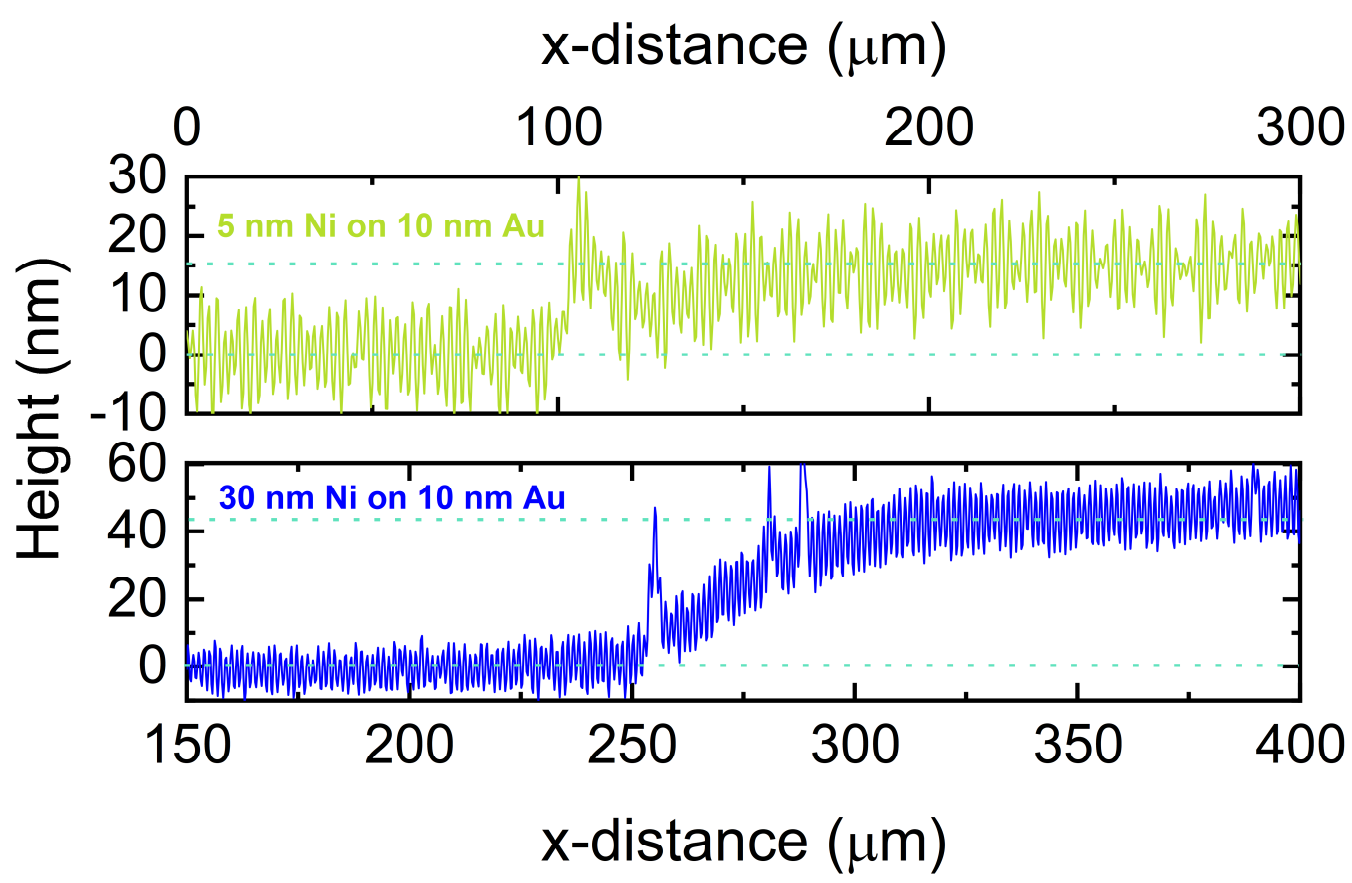

Figure S1 Profilometry of the 5 nm and 30 nm samples deposited on 10 nm Au on top of Si wafer substrate

Profilometry results (**Figure S1**) shows a thickness of ~15.37 nm of the 5 nm Ni + 10 nm Au sample, and ~43.53 nm of the 30 nm Ni + 10 nm Au sample, which is in good agreement with the nominal thickness calculated from the deposition rate. The films also demonstrate a roughness similar to that of the Si wafer substrate, which confirms the flatness of the sample.

3. X-ray photo electron spectroscopy

As seen in the XPS survey spectrum (**Figure S2)**, the sample is free from contaminants.

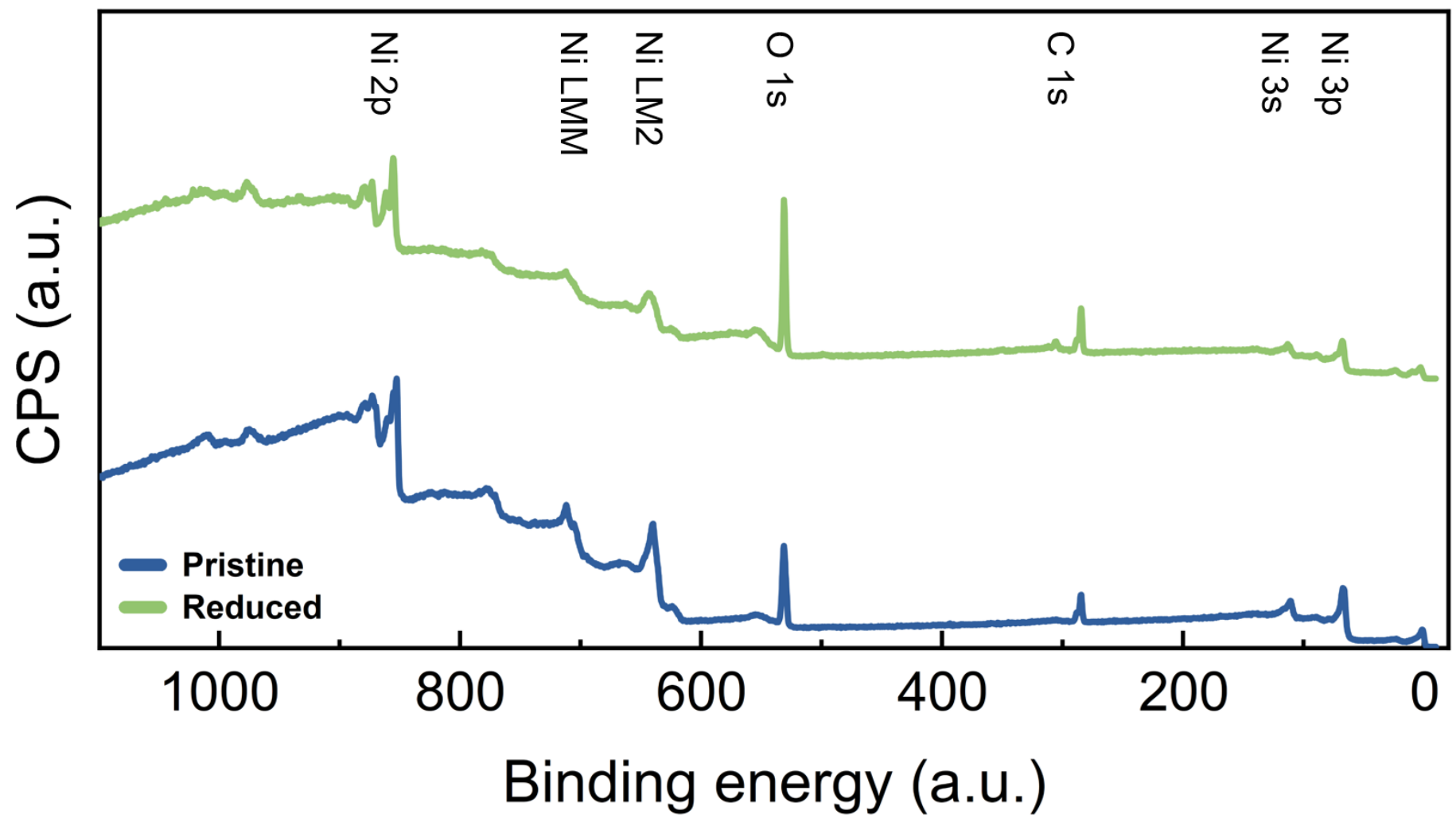

Figure S2 Lab-based XPS (Al K-alpha) survey spectra of the pristine and reduced 30 nm sample

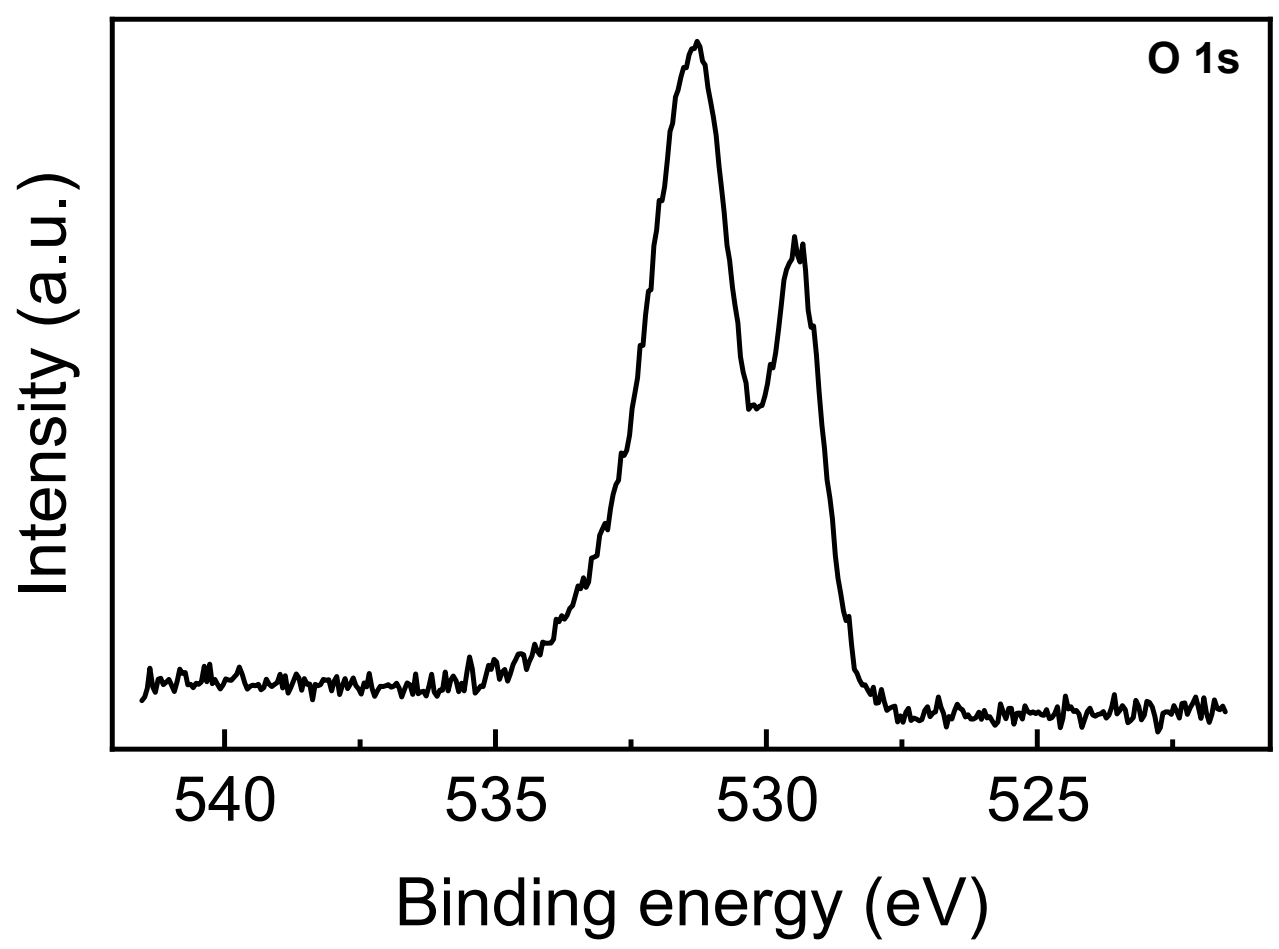

Figure S3 O 1s XPS (1200.0 eV photon energy) spectrum of the 30 nm sample

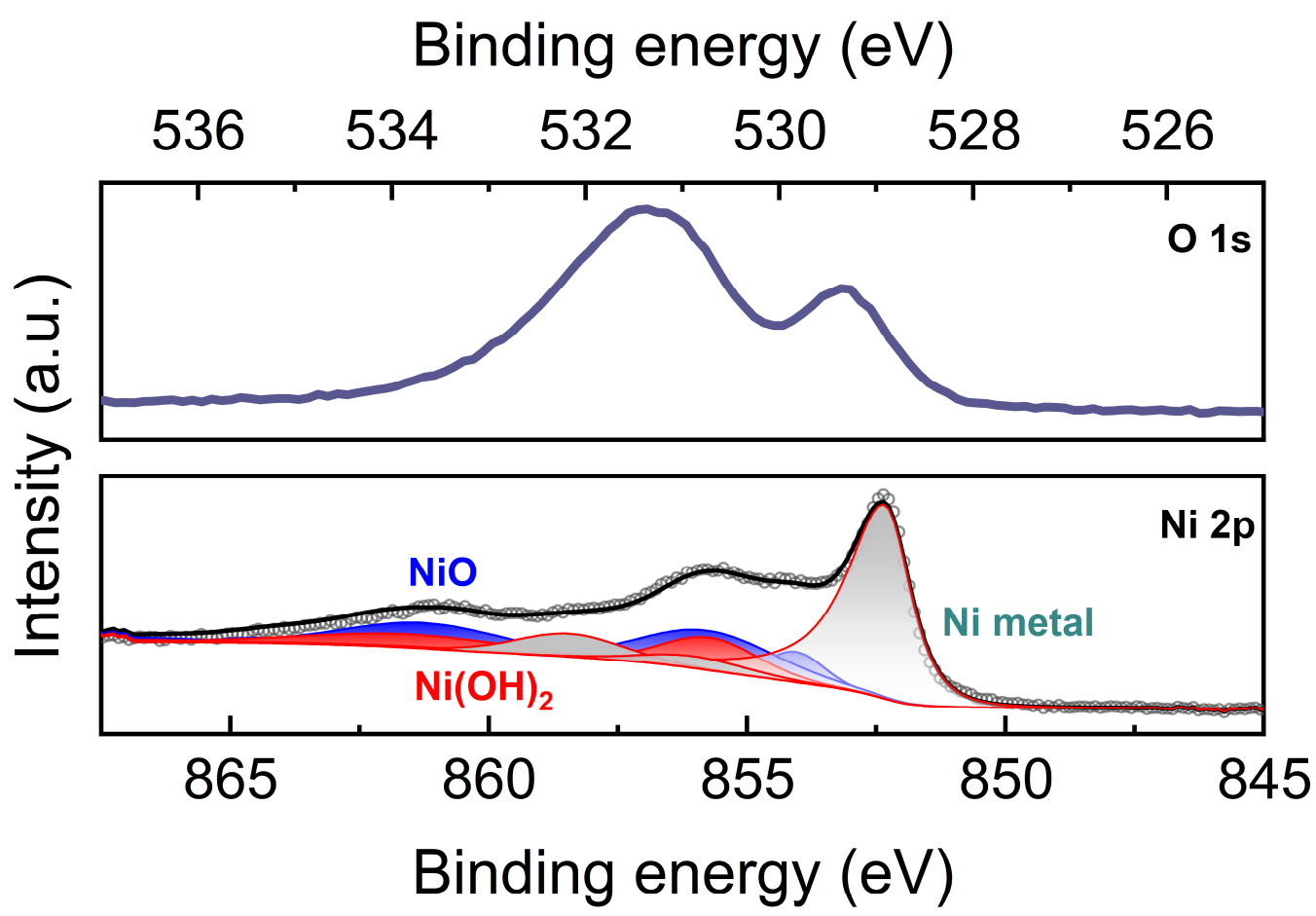

Figure S4 Lab-based XPS (Al K-alpha) O 1s and Ni 2p spectra of the pristine 30 nm sample

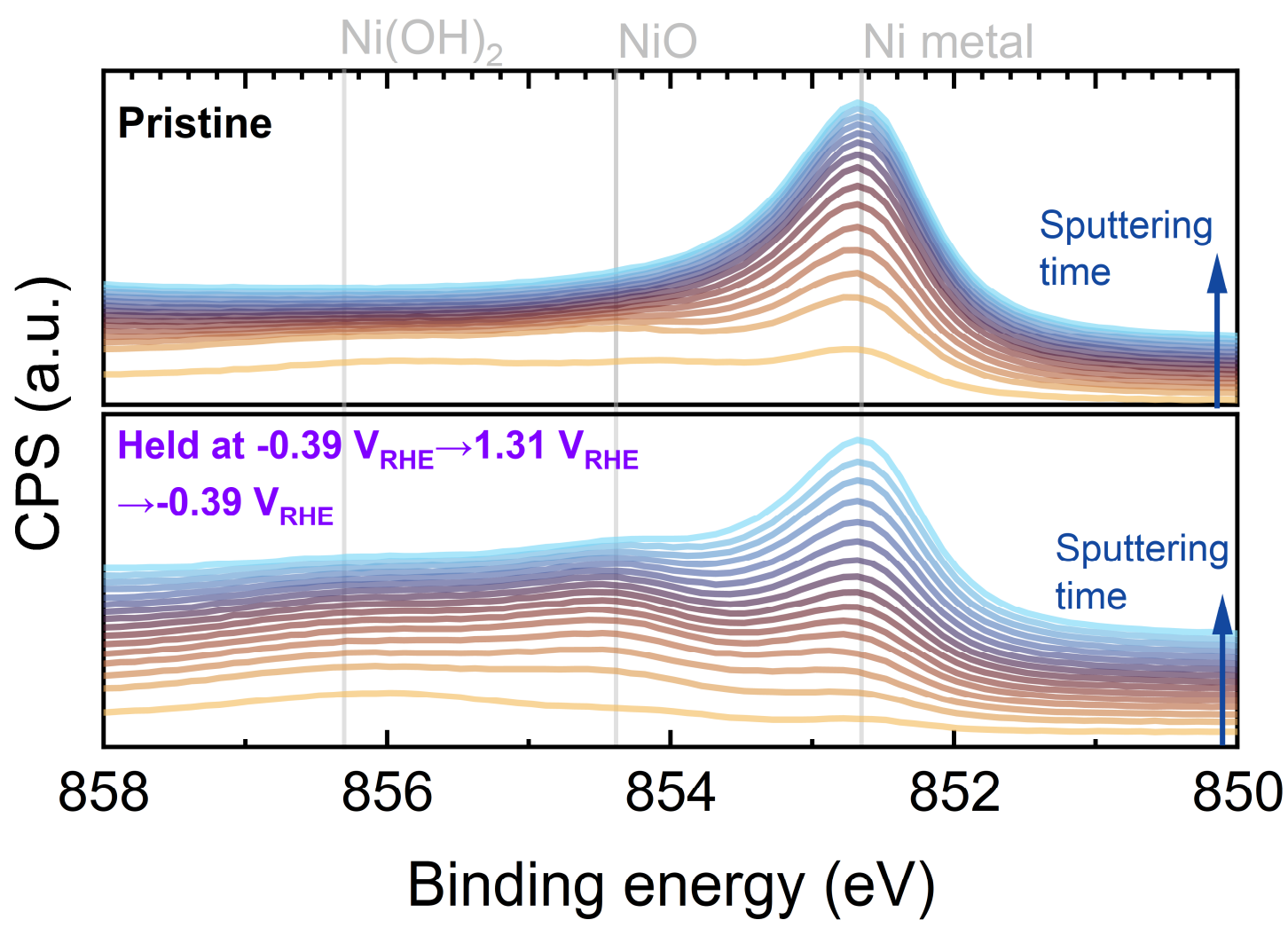

Figure S5 Depth-profiling of the pristine 30 nm sample and sample held at -0.39 V_{RHE} → 1.31 V_{RHE} → -0.39 V_{RHE}. Measurement done on Lab-based XPS.

The Lab-based XPS was measured using a Al K-alpha source (1486.6 eV) energy, which should be more bulk-sensitive compared to the synchrotron XPS (1200 eV). Consequently, we see significantly more contribution from the Ni metal peaks, further confirming that the oxides/hydroxides are confined to the surface.

For the XPS fitting, parameters were taken from Biesinger et al. [5] and is shown in **Table S2** below:

Table S2 Fitting parameters used for the Ni 2p XPS spectra [5]

Peak name	CasaXPS line shape	Position constrains	FWHM
Nickel metal peak 1	LA(1.1,2.2,10)	853.0, 851.8	0.9,1.1
Nickel metal peak 2	LA(1.1,2.2,10)	856.7, 855.5	2.8, 3.1
Nickel metal peak 3	LA(1.1,2.2,10)	859.1, 857.9	2.8, 3.1
NiO peak 1	GL(30)	854.1, 853.5	1.3, 1.4
NiO peak 2	GL(30)	855.8, 855.2	3.1, 3.3
NiO peak 3	GL(30)	861.3, 860.7	3.7, 3.9
NiO peak 4	GL(30)	864.4, 863.8	1.8, 2.1
NiO peak 5	GL(30)	866.7, 866.1	2.3, 2.7
$Ni(OH)_2$ peak 1	GL(30)	856.0, 854.7	0.9, 1.3
$Ni(OH)_2$ peak 2	GL(30)	856.8, 855.5	2.1, 2.4
$Ni(OH)_2$ peak 3	GL(30)	858.8, 857.5	1.5, 1.7
$Ni(OH)_2$ peak 4	GL(30)	861.6, 860.3	1.0, 1.2
$Ni(OH)_2$ peak 5	GL(30)	862.6, 861.3	4.6, 4.7
$Ni(OH)_2$ peak 6	GL(30)	867.6, 866.3	3.0, 3.1

4. Evolution of the nickel cathode at HER potentials

4.1. Unscaled NEXAFS spectra of the Ni standards

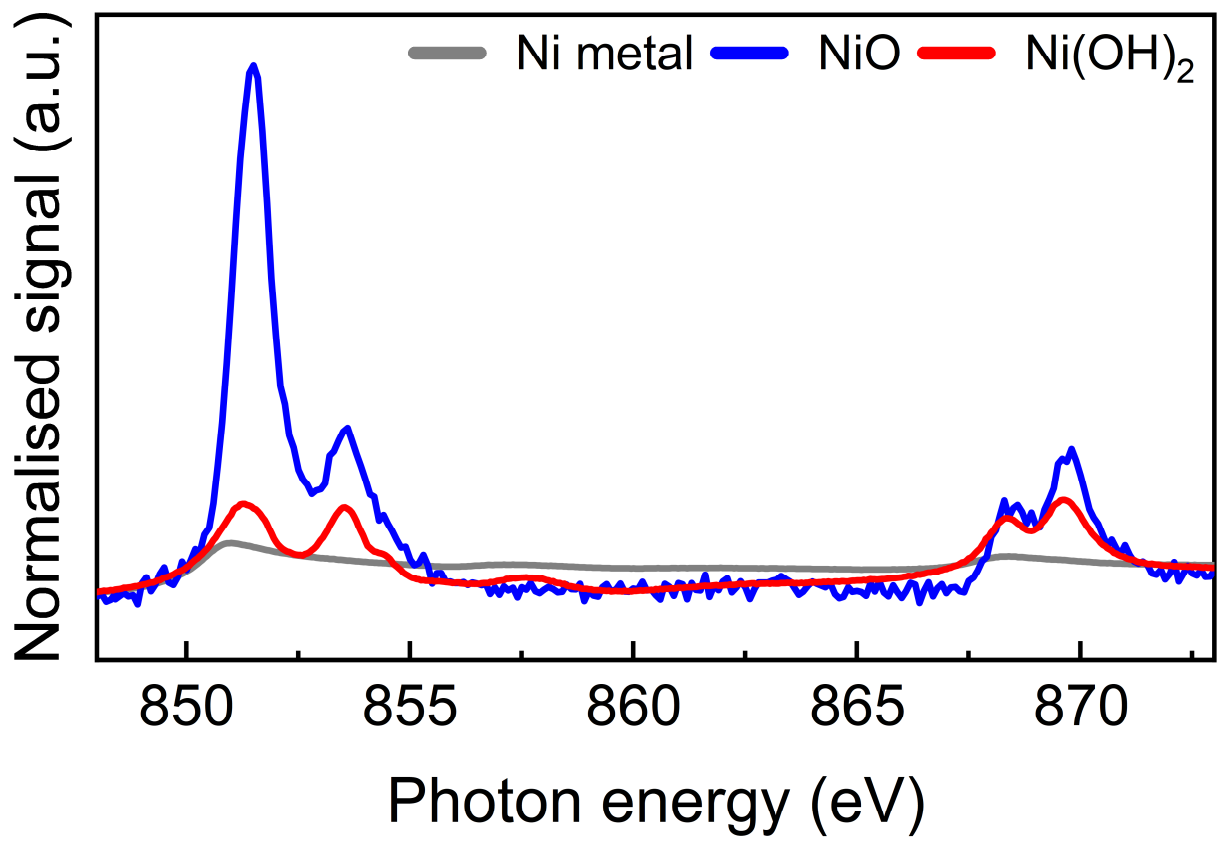

Figure S6 Unscaled, normalised Ni L-edge NEXAFS spectra of Ni metal, NiO and $Ni(OH)_2$

As seen in **Figure S6**, metallic Ni has a much less intense absorption intensity, as well as an edge energy shifted towards lower energy comparing to the oxides. This is accompanied by the absence of peaks at ~853.5 eV and ~854.5 eV for Ni metal. Hence if the catalyst becomes more metallic, it will result in: 1. Reduction in absorption edge intensity; 2. Shift in absorption edge towards lower energy; 3. Reduction in intensity in the features at ~853.5 eV and ~854.5 eV. These features are used to interpret the NEXAFS results in this study and agrees with previous literature [6–9]. An increase in intensity at ~853.5 eV and ~854.5 eV suggests the formation of more oxidised Ni centres. The ratio of the feature at ~853.5 eV relative to ~854.5 eV is higher for NiO compared to $Ni(OH)_2$.

4.2 Reduced scan rate CV of the pristine 30 nm sample

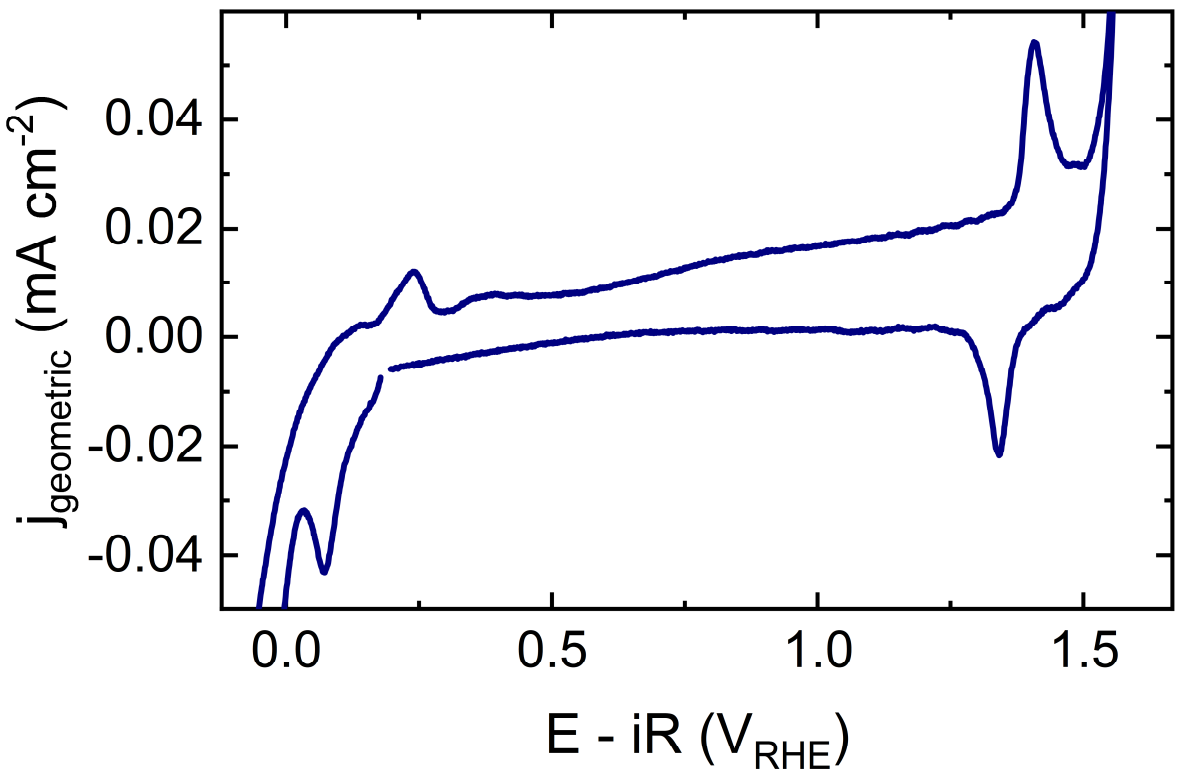

Figure S7 Cyclic voltammogram (CV) done at 5 mV/s of the pristine 30 nm sample. Measurements were done in 0.1 M Ar-saturated KOH, 1600 rpm, room temperature and pressure

4.3 Online EC-MS measurements

Calibration of the online EC-MS was done using a polished Pt RDE stub in 0.1 M $HClO_4$ with Ar as carrier gas. The chip-based EC–MS system used here (SpectroInlets) has been shown to have a response time of ~1.7 s. [10] Here, the time step has been set to 0.7 s. Hence, we do not expect the conclusion to be affected by response time.

The amount of charged passed during a certain amount of time can integrated from the current with time:

$$Q_{H_2,Pt} = \int j_{measured,Pt}\, dt$$

Where $Q_{H_2,Pt}$ is the total charge used to produced H_2 on Pt in C cm^{-2}, and $j_{measured,Pt}$ is the geometric current density measured in A cm^{-2} or C s^{-1} cm^{-2}. Assuming the Faradic efficiency on Pt to be 100% and 1 mol of H_2 requires 2 mol of electrons to form, the $Q_{H_2,Pt}$ in C cm^{-2} can be

converted into the amount of H_2 produced in mol cm^{-2}, assuming the faradic efficiency on Pt to be 100%:

$$n_{H_2} = \frac{Q_{H_2,Pt}}{2 \times N_A \times e}$$

Where n_{H_2} is the amount of H_2 produced over time in mol cm^{-2}, N_A is the Avogadro's constant, and e is the elementary charge which is 1.6×10^{-19} C. Differentiating the above equation with time yields:

$$\frac{\delta n_{H_2}}{\delta t} = \frac{1}{2 \times N_A \times e} \times \frac{\delta Q_{H_2,Pt}}{\delta t} = \frac{1}{2 \times N_A \times e} \times j_{measured}$$

Which connects the rate of H_2 production in mol s^{-1} cm^{-2} with the current measured in A cm^{-2} or C s^{-1} cm^{-2}.

The EC-MS records H_2 signal in counts s^{-1}, which can also be integrated and plotted against the amount of H_2 calculated from the current density (n_{H_2} in mol cm^{-2}) to achieve a calibration curve (**Figure S8**), giving a 0.0247 counts mol^{-1} cm^2 calibration value.

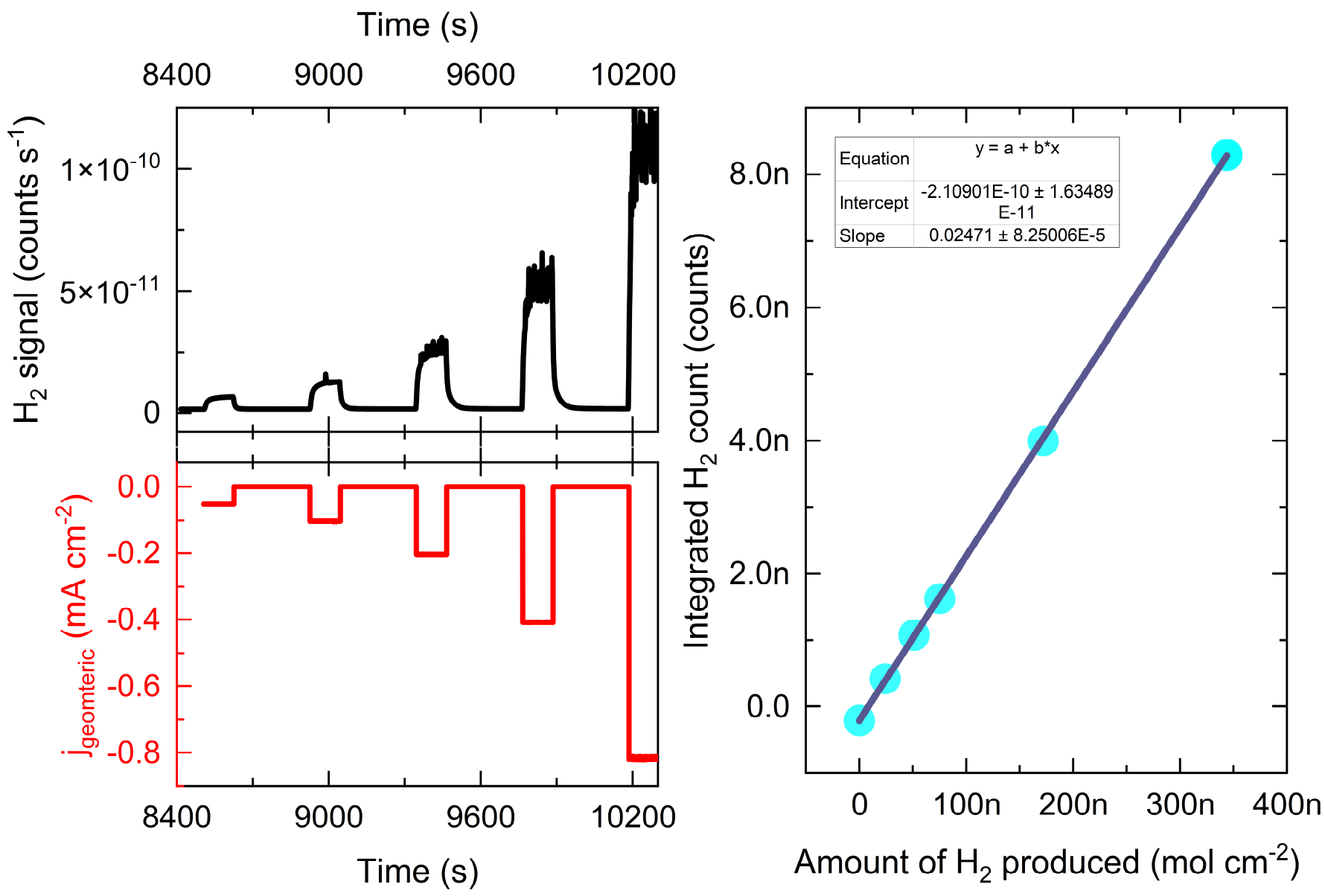

Figure S8 Calibration of the online EC-MS system using a Pt RDE stub; n = 10^{-9}.

In the case of Ni catalysts, as discussed in the main text, the HER process convolutes with the reduction of the oxides. Assuming HER and reduction of NiO_xH_y are the only two processes happening, the total charge pass onto the system can be decomposed into:

$$Q_{total} = \int j_{measured}\, dt = Q_{HER} + Q_{oxide\ reduction}$$

Where Q_{total} is the total charge integrated from the total measured current $j_{measured}$. The part of charge used to produce H_2 (Q_{HER}, C cm^{-2}) from the total current on Ni catalysts (Q_{total}) can be calculated from the H_2 counts from the EC-MS as:

$$Q_{HER} = \frac{\int H_2\ counts\ dt}{0.02471} * 2 * N_A * e$$

And the faradic efficiency can be calculated as:

$$FE = \frac{Q_{HER}}{Q_{total}}$$

It is worth noting that the calculations assume no non-faradic processes such as charging occurs during the process.

4.4 Evolution of NEXAFS spectra under HER conditions

As shown in **Figure S9**, the spectrum obtained at open circuit potential (OCP) for the 30 nm film is dominated by water signal [2], which implies the whole sample is within probing range.

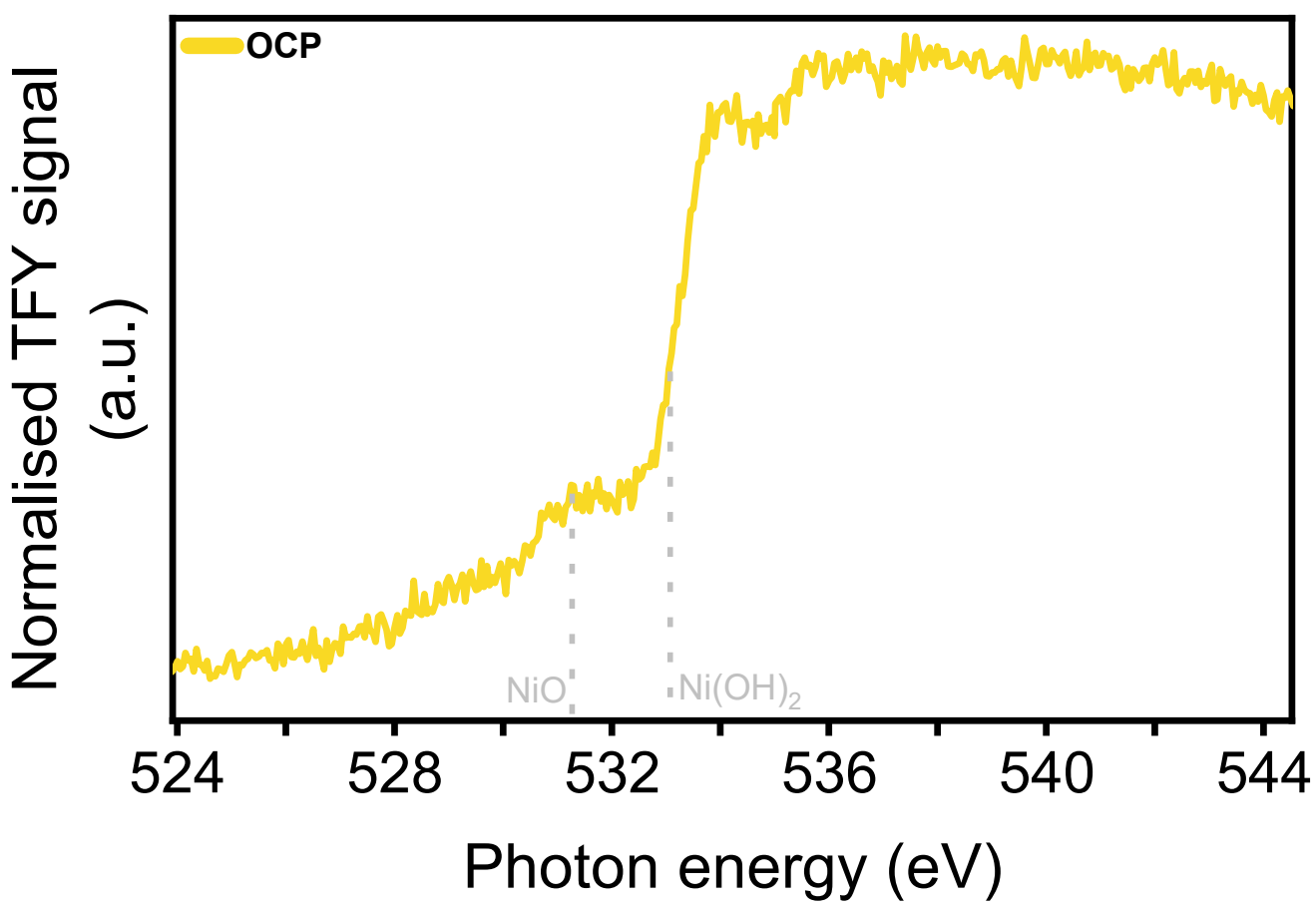

Figure S9 O K-edge spectrum taken for the 30 nm pristine film at open circuit potential (OCP)

For easier comparison, the position of Ni L3 edge is plotted against the potentials applied in **Figure 4** as shown in **Figure S10** below.

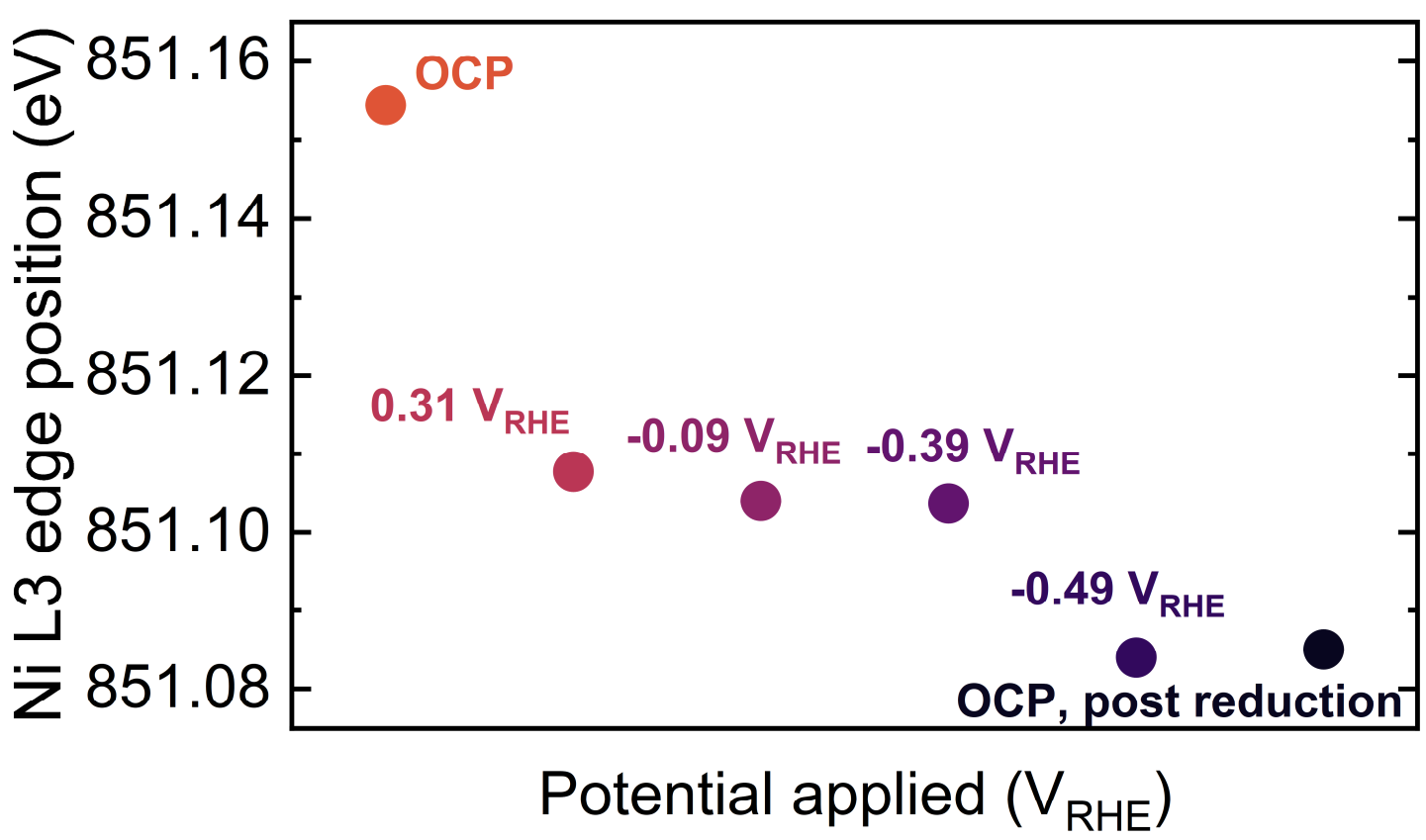

Figure S10 Change in position in the peak of the Ni L3 edge spectra as a function of potential during the reduction process.

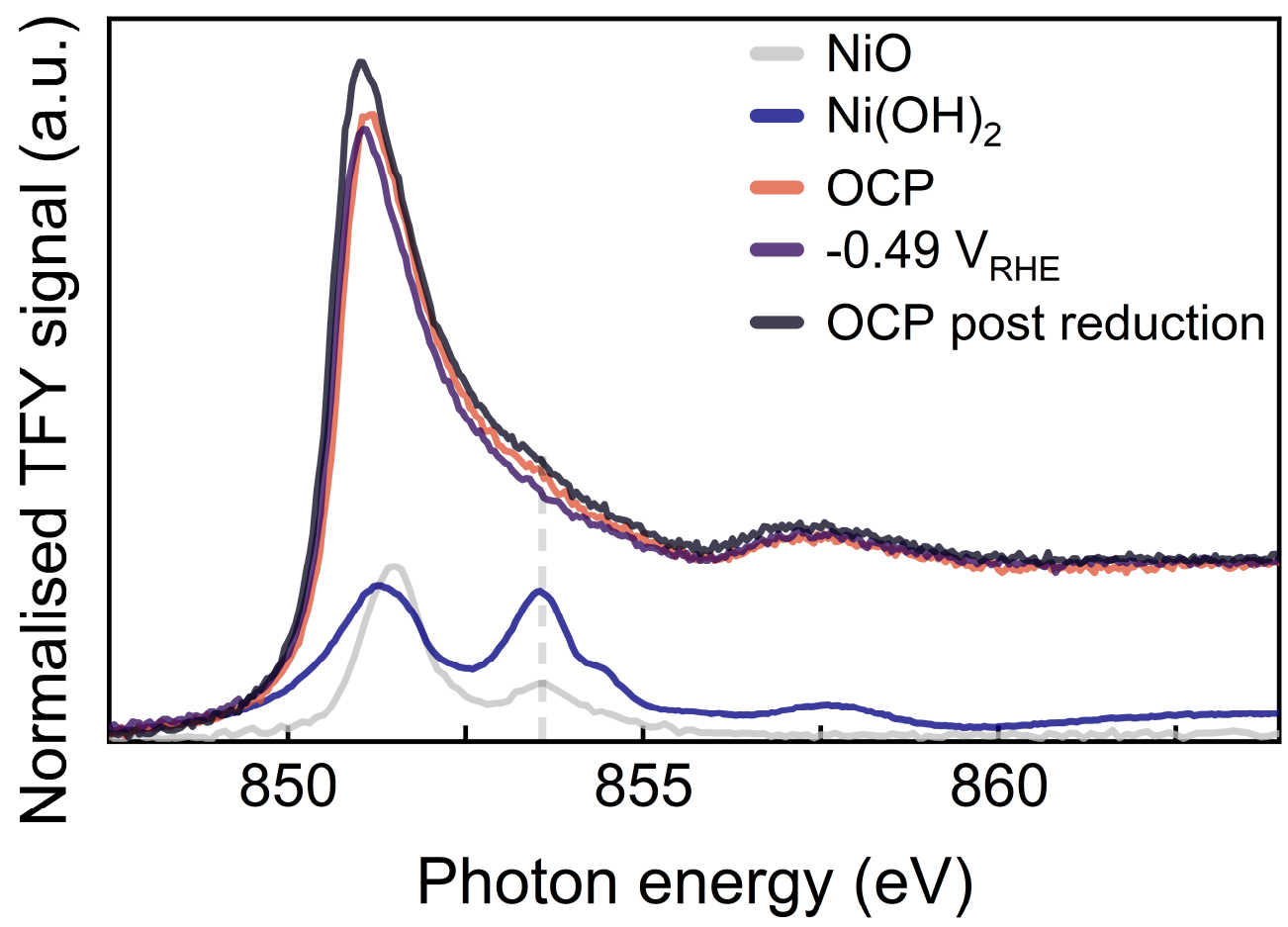

Figure S11 Overlapped *operando* Ni L-edge NEXAFS spectra at OCP, during reduction (-0.49 V_{RHE}) and at OCP post reduction. The standards spectra are scaled.

5. Extra plots of the NanoSIMS measurements

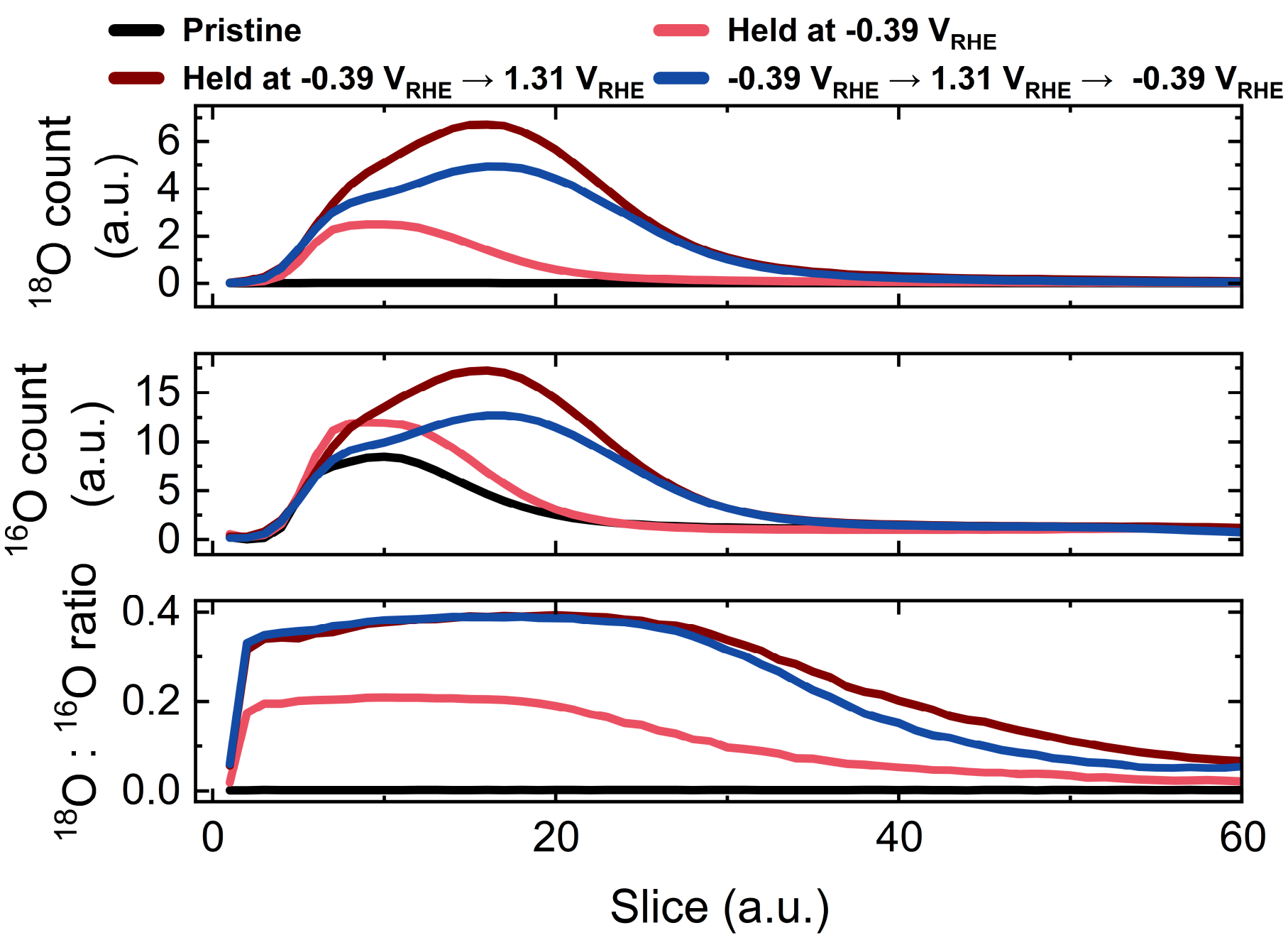

Figure S12 NanoSIMS depth profiles of (i) the pristine 30 nm Ni film and the sample after (ii) holding at −0.39 V_{RHE} (pink), (iii) holding at −0.39 V_{RHE} followed by oxidation at 1.31 V_{RHE} (brown), and (iv) holding at −0.39 V_{RHE} followed by oxidation at 1.31 V_{RHE} and reduction at −0.39 V_{RHE} (blue).

6. Role of oxidised Ni species in modulating HER activity

6.1 Sustained enhancement of activity

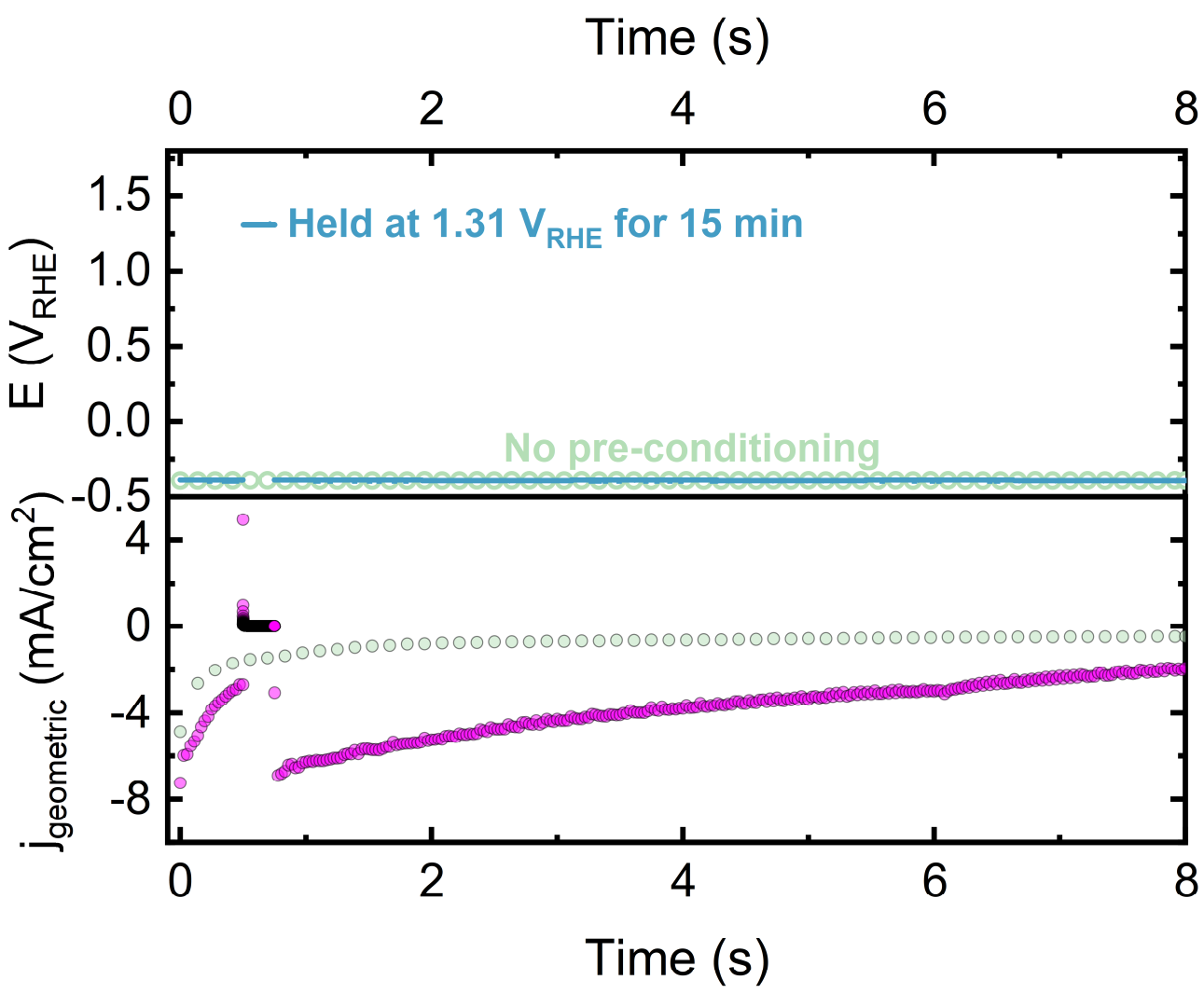

Figure S13 Chronoamperometric (CA) response of 30 nm Ni films during a potential hold at −0.39 V_{RHE} for 30 minutes, followed by anodic oxidation at 1.31 V_{RHE} for 15 minutes and subsequent reduction at −0.39 V_{RHE} for 7 hours and 15 minutes. CA without pre-conditioning (held at at −0.39 V_{RHE} for 8 hours) is shown in light green hollow circles.

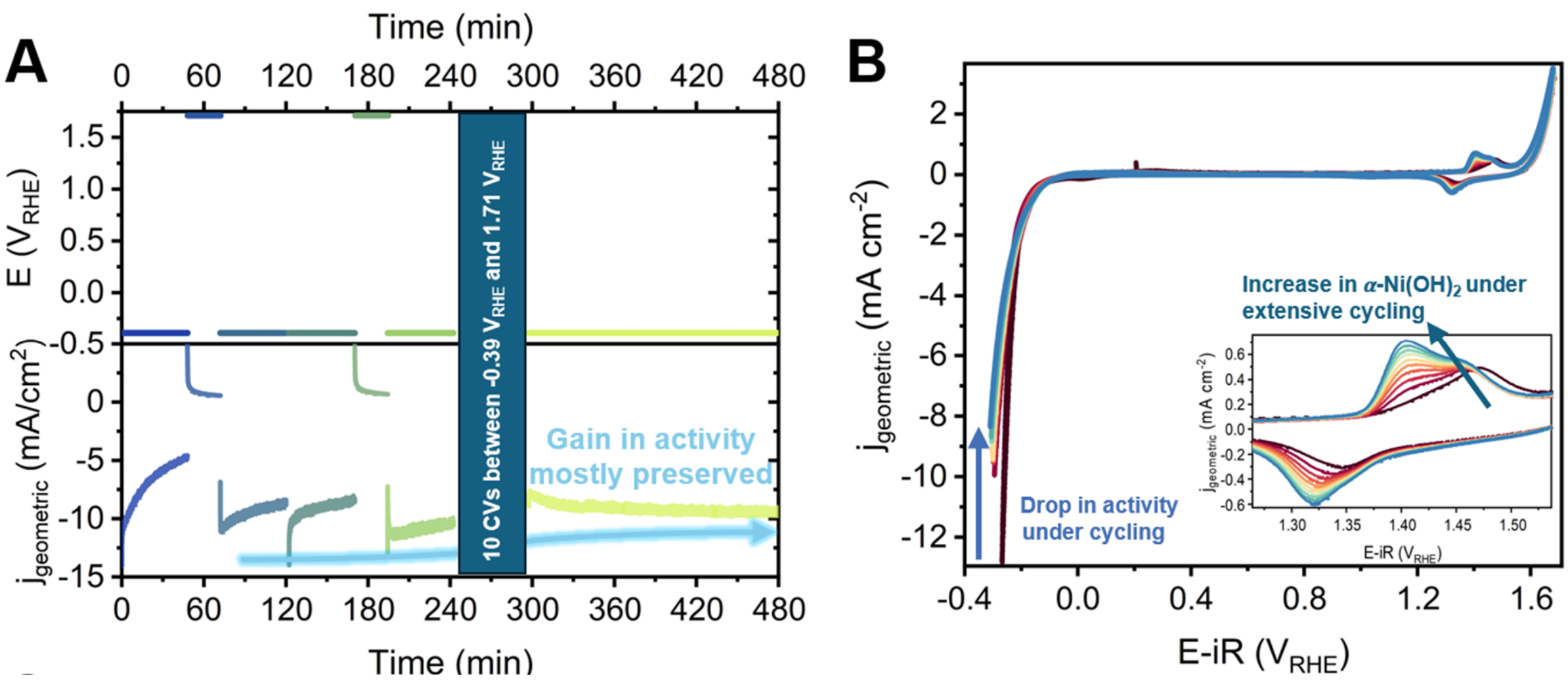

Figure S14 A: current density and potential applied overtime for the extended time experiment on the 30 nm film; **B:** The 10 cycles of cyclic voltammogram (CV) conducted

6.2 Comparison between films with different thicknesses

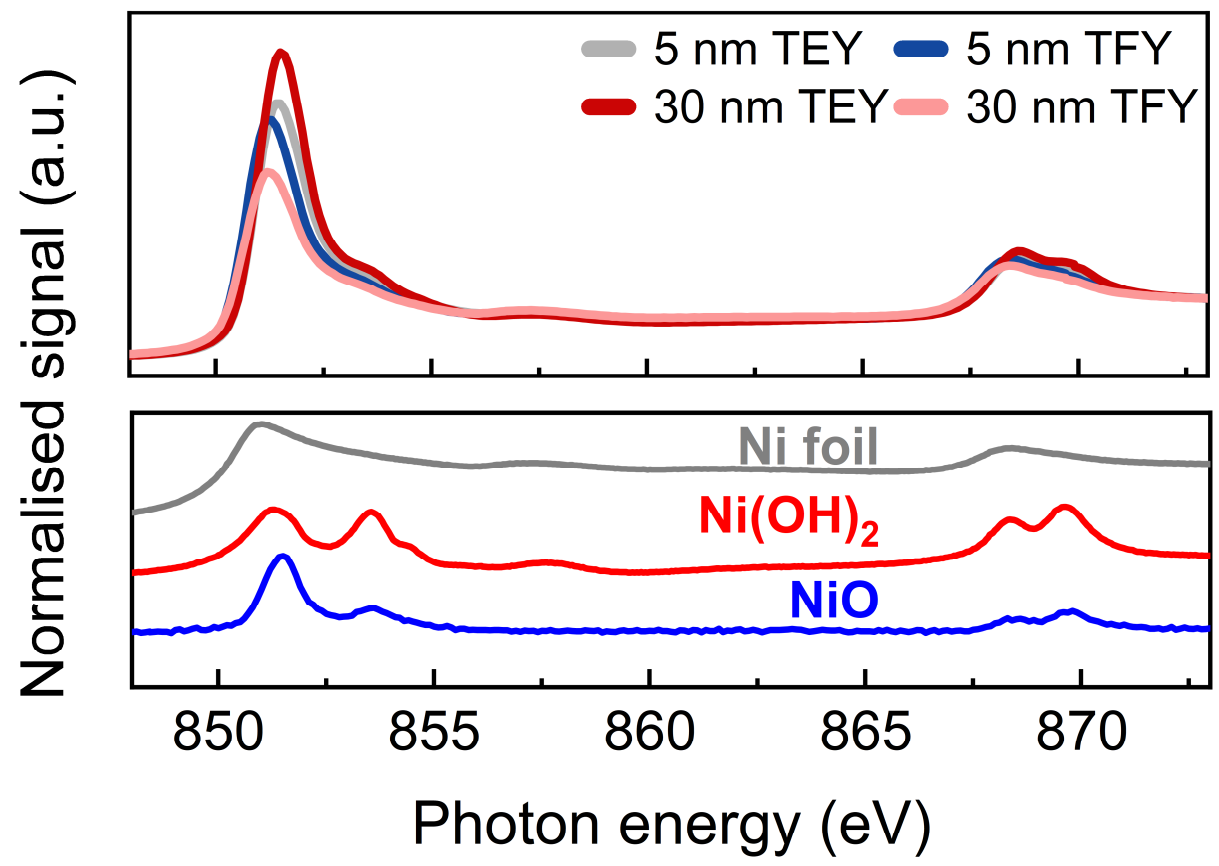

Figure S15: Comparison of Ni L-edge near edge X-ray absorption fine structure **(**NEXAFS) spectra of the 30 nm vs 5 nm pristine films. Standards spectra are scaled for visibility.

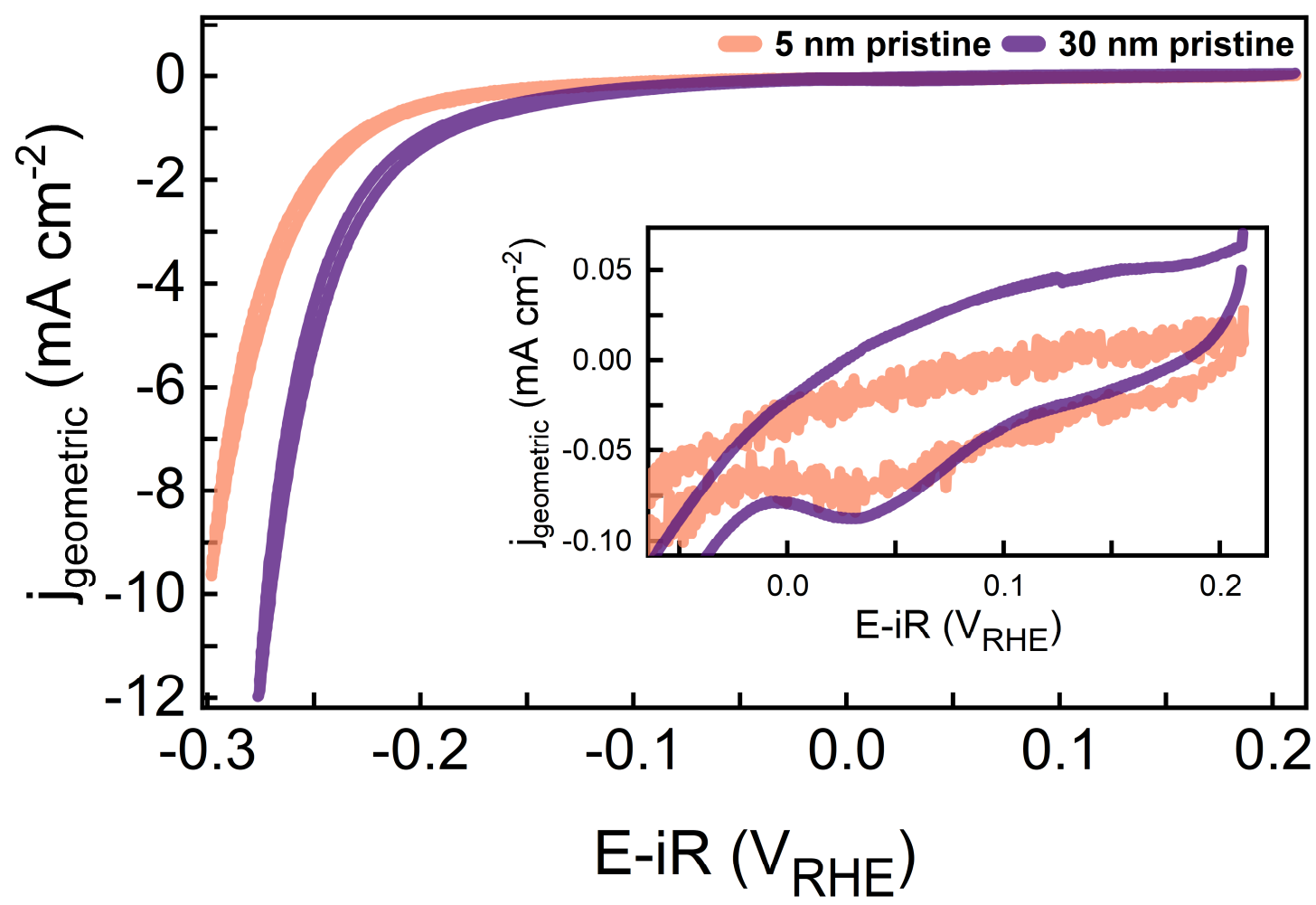

Figure S16: Comparison of the cyclic voltammetry and the capacitive current region for pristine 30 nm and 5 nm thin film.

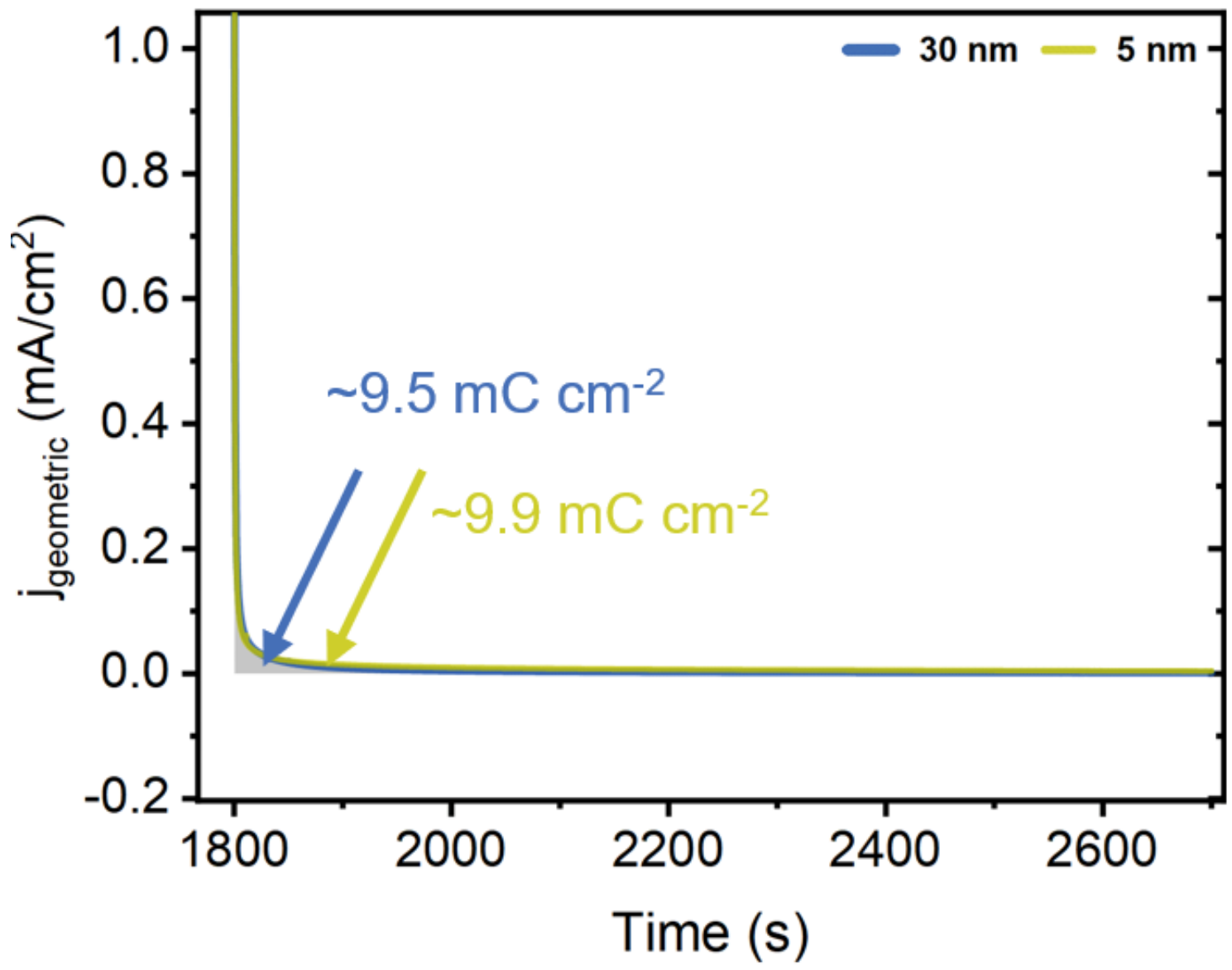

Figure S17 Integrated charge for 30 nm and 5 nm film during the oxidation step at 1.31 V_{RHE}

As shown in **Figure S18**, the $Ni(OH)_2 + OH^- \rightarrow NiOOH + H_2O + e^-$ redox wave can be integrated to compare the charge passed during the redox for both films. The integrated charges are comparable between both thicknesses.

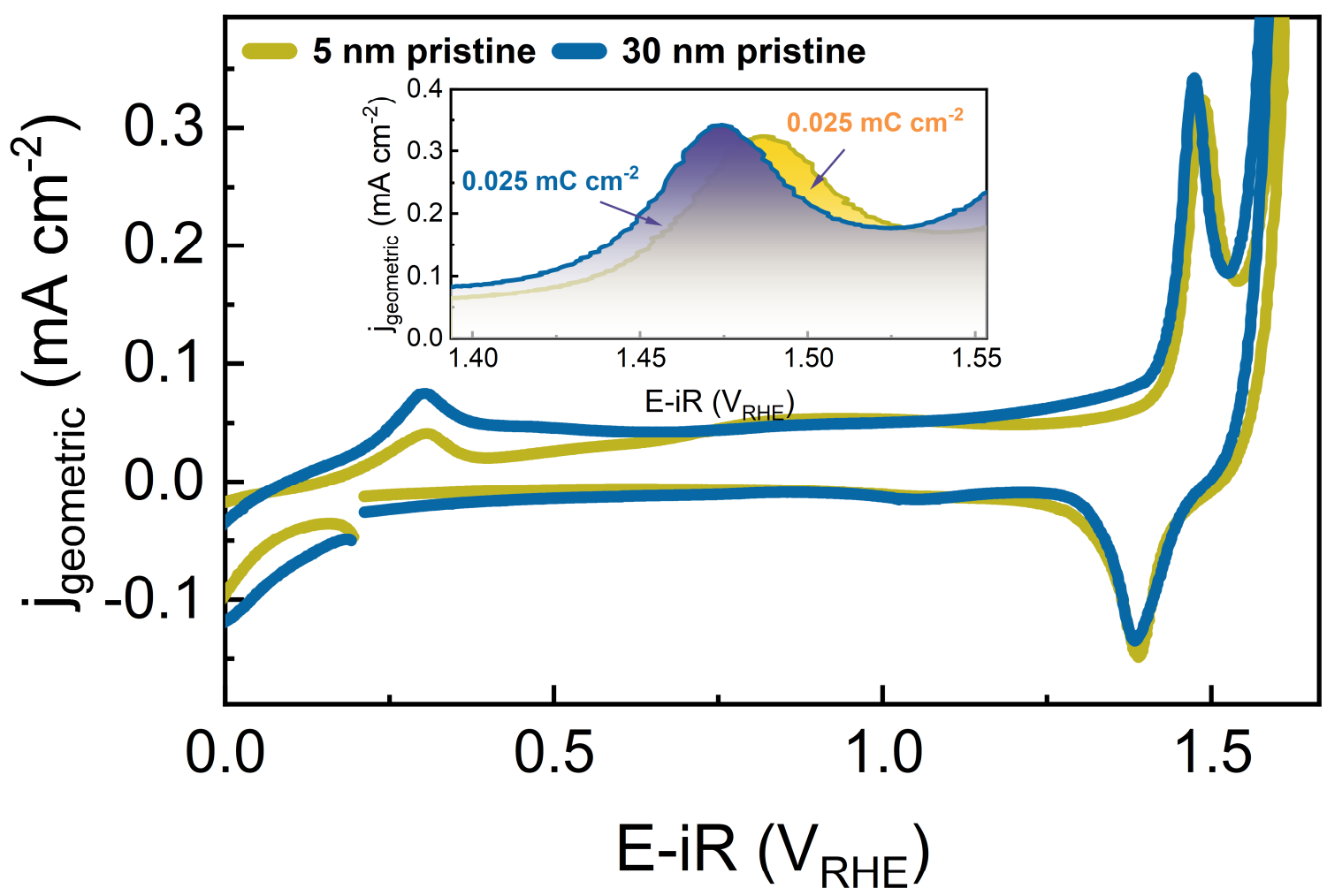

Figure S18 – Comparable oxidation charges for the pristine 5 nm and 30 nm. 20 mV/s Cyclic voltammogram (CV) done in Ar-saturated 0.1 M KOH using a Hg/HgO reference electrode and a carbon rod counter electrode.

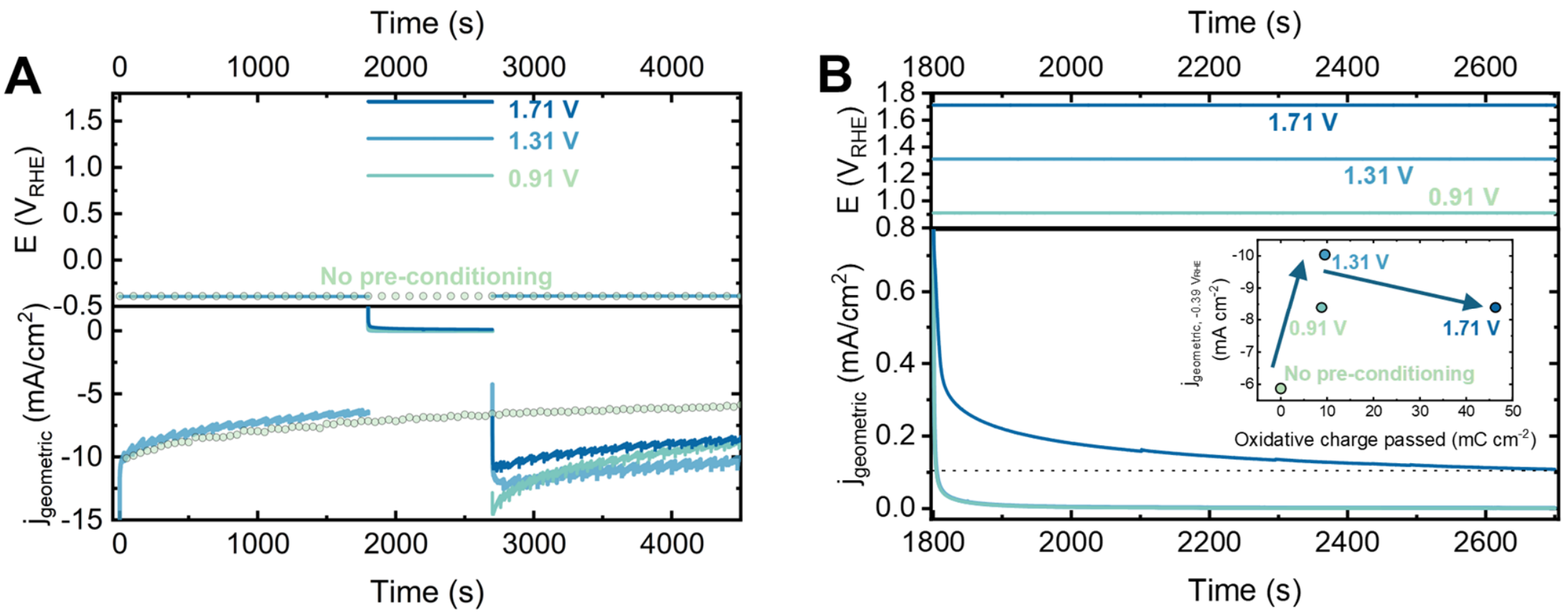

Figure S19 A: Chronoamperometric response of 30 nm Ni films hold at −0.39 V_{RHE} for 30 minutes, followed by anodic oxidation at 0.91, 1.31 and 1.71 V_{RHE} for 15 minutes respectively, and subsequent reduction at −0.39 V_{RHE} for 30 minutes. Sample without any preconditioning is plotted in circles; ***B*:** Integration of oxidative charges during different pre-conditioning potentials and the corresponding current density at subsequent hydrogen evolution potential (-0.39 V_{RHE}).The current densities recorded in the and subsequent reduction at −0.39 V_{RHE} at 30 minutes for sample without pre-conditioning and sample held at different anodic oxidation potentials. The black dashed line was used as the baseline of integration as the potential applied was well into the OER region (1.71 V_{RHE}).

6.2 Extra spectroscopic data

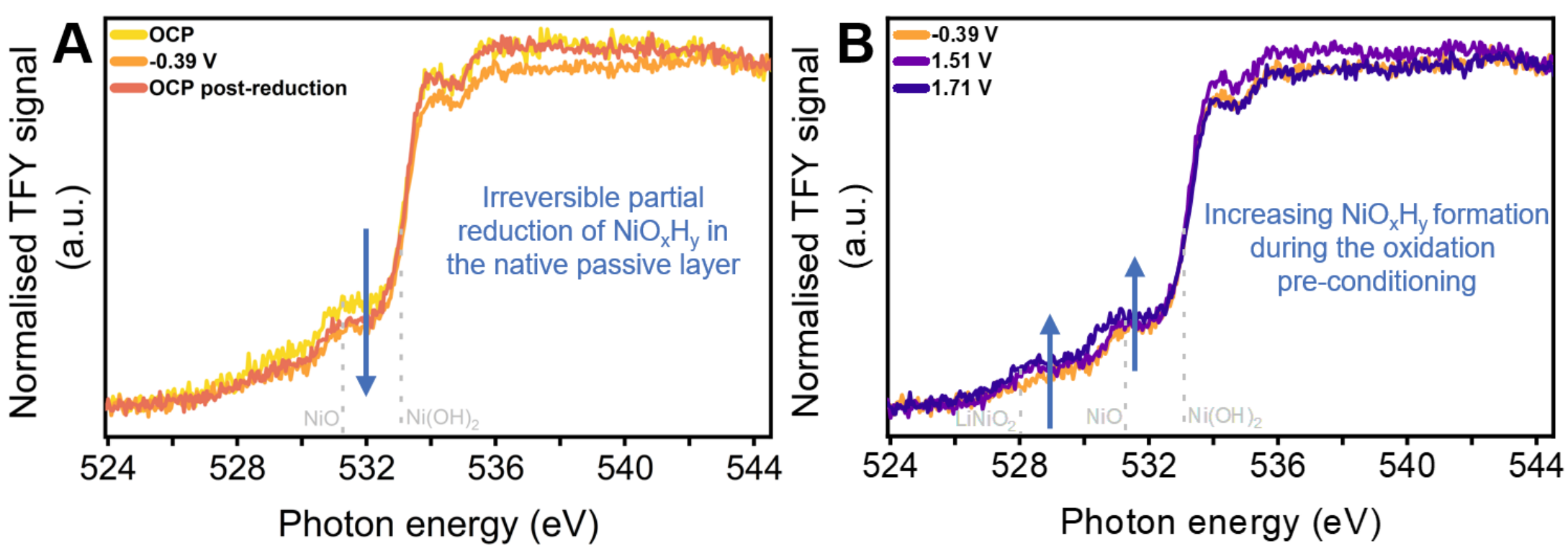

Figure S20 *Operando* near edge X-ray absorption fine structure (NEXAFS) collected for O K-edge on the 30 nm Ni film, **A:** during reduction; **B:** during oxidation.

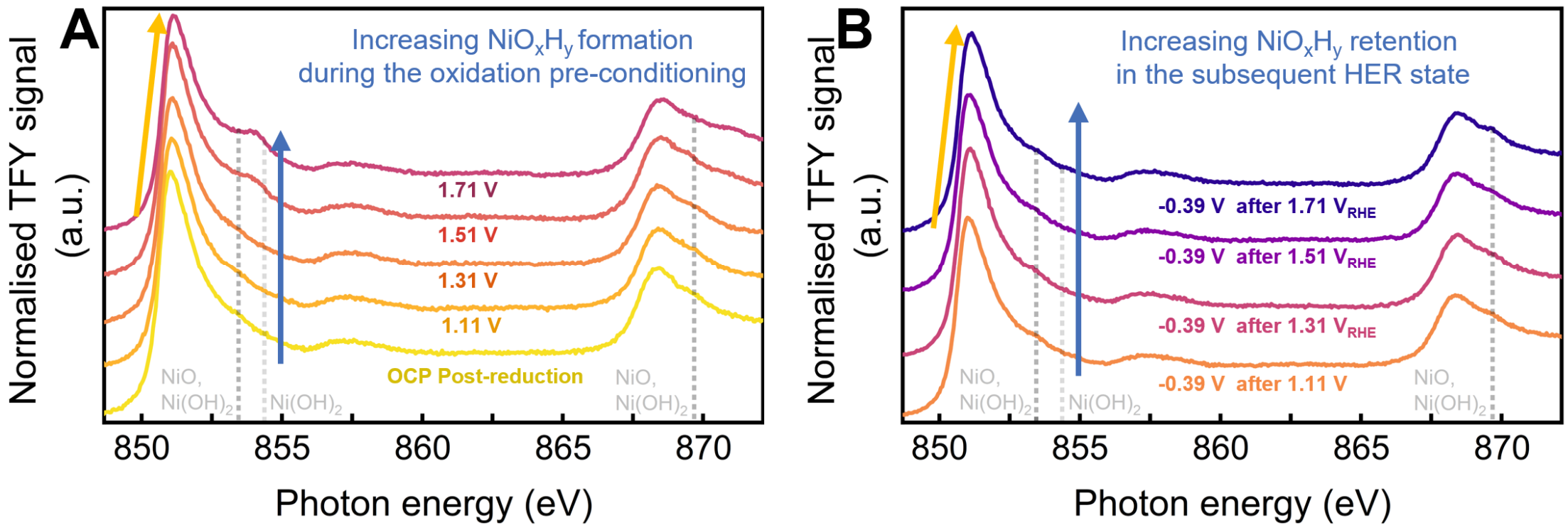

Figure S21 *Operando* near edge X-ray absorption fine structure (NEXAFS) spectra collected for Ni L-edge on the 30 nm Ni film, **A:** under oxidative potentials; **B:** during the subsequent reduction potential after pre-conditioning at oxidative potentials.

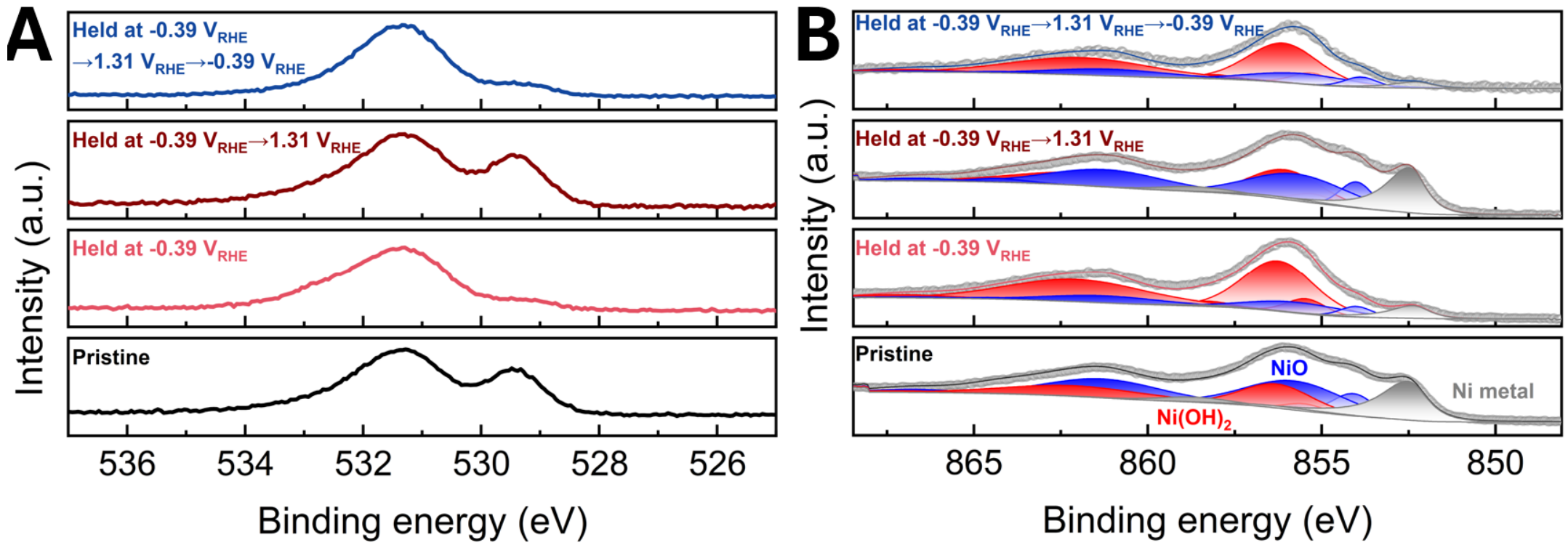

Figure S22 X-Ray Photoelectron Spectroscopy (XPS, 1200.0 eV photon energy) spectra of O 1s (A) and Ni 2p spectra (B) of the 30 nm sample measured under different conditions

6.3 Kinetic analysis

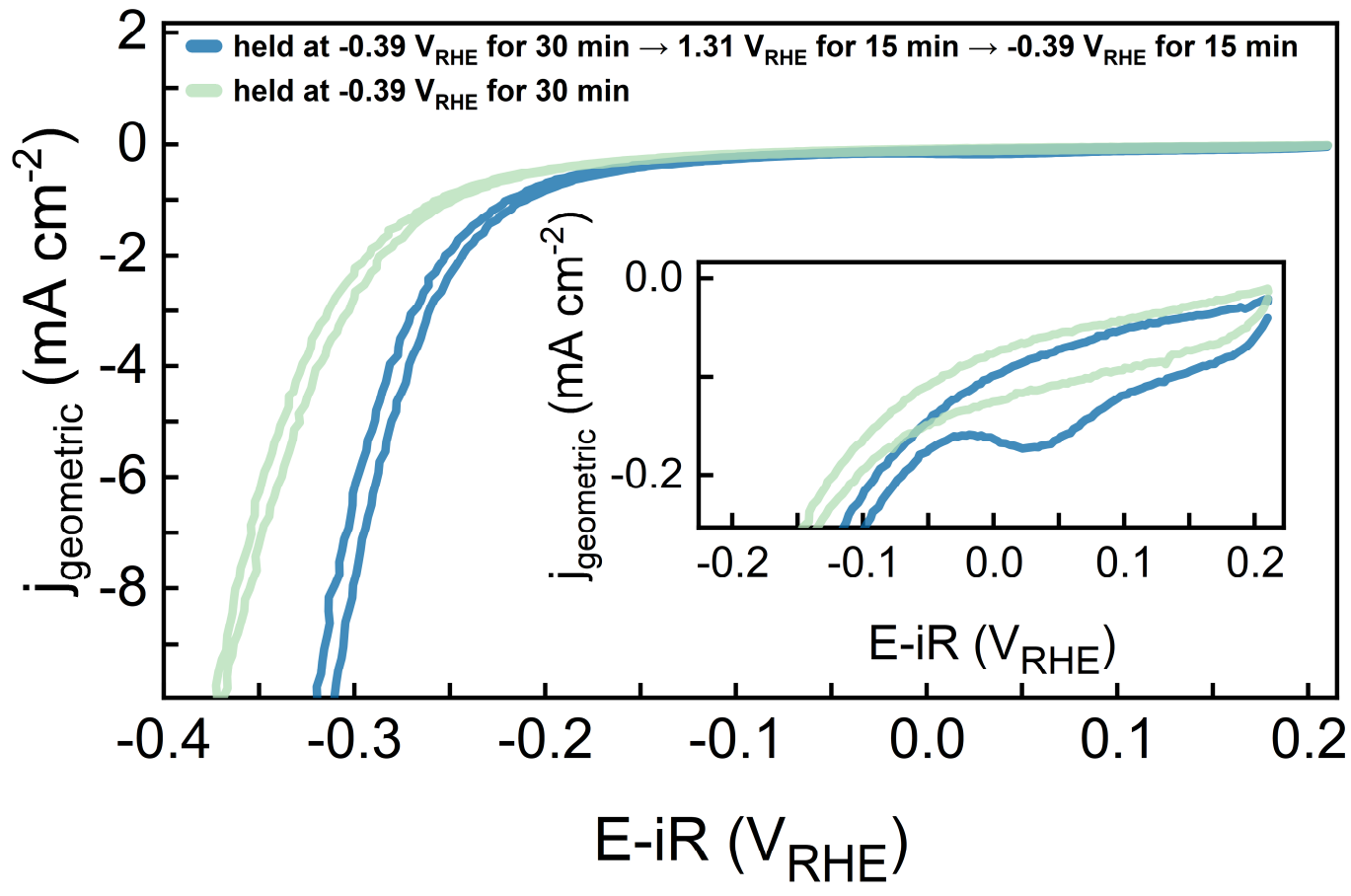

Figure S23 – CV of the 5 nm sample held at -0.39 V_{RHE} for 30 minutes → 1.31 V_{RHE} for 15 minutes → -0.39 V_{RHE} for 30 minutes (blue), and the sample held at -0.39 V_{RHE} for 30 minutes (light green). Inset shows the redox peak region.

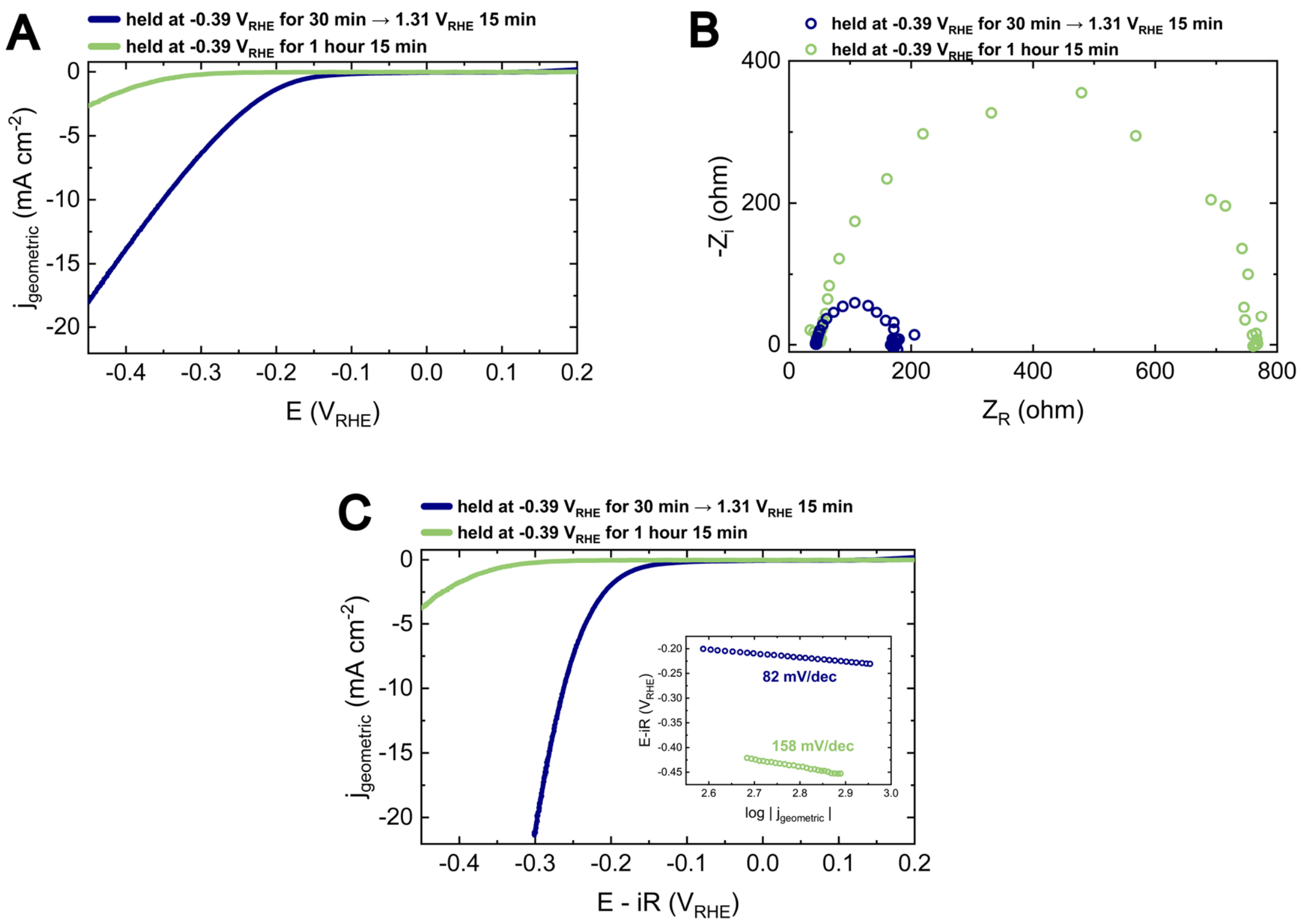

Figure S24 Kinetic analysis on the 5 nm sample. **A:** Linear sweep voltammogram (LSV) of the sample done at 5 mV/s, showing the sample held at -0.39 V_{RHE} for 30 minutes then oxidised at 1.31 V_{RHE} for 15 minutes (blue), and the sample held at -0.39 V_{RHE} for 1 hour 15 minutes (green); **B:** Nyquist plot of impedance data measured at -0.39 V_{RHE} (uncorrected potential); **C:** 100% iR-corrected LSV and the corresponding Tafel slope analysis (inset); Measurements were done in 0.1 M Ar-saturated KOH, 1600 rpm, room temperature and pressure, carbon rod counter electrode, Hg/HgO reference electrode.

6.4 Extra SEM images

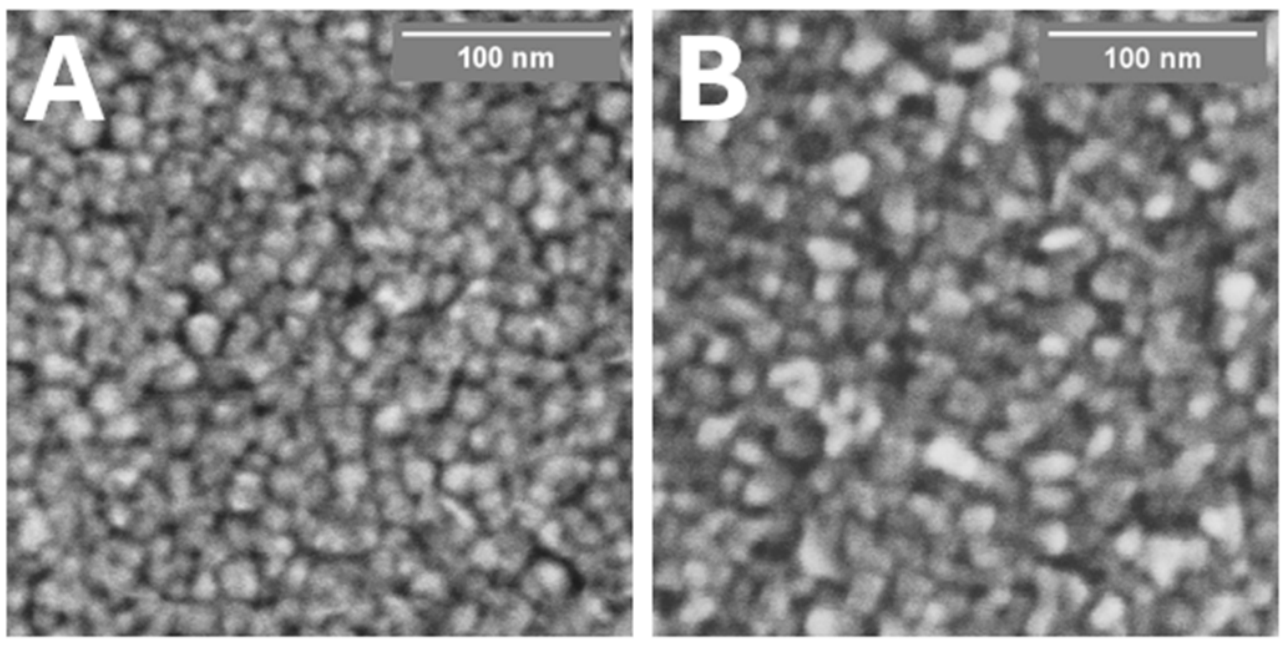

Figure S25 – Scanning electron microscopy (SEM) images of **A:** the 30 nm sample held at -0.39 V_{RHE} and **B:** the 30 nm sample held at -0.39 V_{RHE}, followed by anodic oxidation at 1.31 V_{RHE} then -0.39 V_{RHE}.

REFERENCES

(1) Dent, A. J.; Cibin, G.; Ramos, S.; Smith, A. D.; Scott, S. M.; Varandas, L.; Pearson, M. R.; Krumpa, N. A.; Jones, C. P.; Robbins, P. E. B18: A Core XAS Spectroscopy Beamline for Diamond. *J. Phys. Conf. Ser.* **2009**, *190* (1), 012039. https://doi.org/10.1088/1742-6596/190/1/012039.

(2) Grinter, D. C.; Ferrer, P.; Venturini, F.; Spronsen, M. A. van; Large, A. I.; Kumar, S.; Jaugstetter, M.; Iordachescu, A.; Watts, A.; Schroeder, S. L. M.; Kroner, A.; Grillo, F.; Francis, S. M.; Webb, P. B.; Hand, M.; Walters, A.; Hillman, M.; Held, G. VerSoX B07-B: A High-Throughput XPS and Ambient Pressure NEXAFS Beamline at Diamond Light Source. *J. Synchrotron Radiat.* **2024**, *31* (3), 578–589. https://doi.org/10.1107/S1600577524001346.

(3) Justin Gorham. NIST X-Ray Photoelectron Spectroscopy Database - SRD 20, 2012. https://doi.org/10.18434/T4T88K.

(4) Schroeder, S. L. M.; Moggridge, G. D.; Ormerod, R. M.; Rayment, T.; Lambert, R. M. What Determines the Probing Depth of Electron Yield XAS? *Surf. Sci.* **1995**, *324* (2), L371–L377. https://doi.org/10.1016/0039-6028(94)00779-9.

(5) Biesinger, M. C.; Payne, B. P.; Grosvenor, A. P.; Lau, L. W. M.; Gerson, A. R.; Smart, R. St. C. Resolving Surface Chemical States in XPS Analysis of First Row Transition Metals, Oxides and Hydroxides: Cr, Mn, Fe, Co and Ni. *Appl. Surf. Sci.* **2011**, *257* (7), 2717–2730. https://doi.org/10.1016/j.apsusc.2010.10.051.

(6) Wang, H.; Butorin, S. M.; Young, A. T.; Guo, J. Nickel Oxidation States and Spin States of Bioinorganic Complexes from Nickel L-Edge X-Ray Absorption and Resonant Inelastic X-Ray Scattering. *J. Phys. Chem. C* **2013**, *117* (47), 24767–24772. https://doi.org/10.1021/jp402404b.

(7) Wang, H.; Patil, D. S.; Gu, W.; Jacquamet, L.; Friedrich, S.; Funk, T.; Cramer, S. P. L-Edge X-Ray Absorption Spectroscopy of Some Ni Enzymes: Probe of Ni Electronic Structure. *J. Electron Spectrosc. Relat. Phenom.* **2001**, *114–116*, 855–863. https://doi.org/10.1016/S0368-2048(00)00370-4.

(8) Gu, W.; Wang, H.; Wang, K. Nickel L-Edge and K-Edge X-Ray Absorption Spectroscopy of Non-Innocent Ni[S2C2(CF3)2]2n Series (n = −2, −1, 0): Direct Probe of Nickel Fractional Oxidation State Changes. *Dalton Trans.* **2014**, *43* (17), 6406–6413. https://doi.org/10.1039/C4DT00308J.

(9) Yu, Y.; Wang, Y.; Ye, H.; Halder, S.; Yang, G.; Ye, B.; Kumar, S.; Held, G.; Durrant, J. R.; Titirici, M.-M.; Rao, R. R. Mechanistic Study of Glycerol Electro-Oxidation on Ni(OH)2/NiOOH Electrodes. *J. Am. Chem. Soc.* **2026**, *148* (15), 16119–16128. https://doi.org/10.1021/jacs.6c00726.

(10) Trimarco, D. B.; Scott, S. B.; Thilsted, A. H.; Pan, J. Y.; Pedersen, T.; Hansen, O.; Chorkendorff, I.; Vesborg, P. C. K. Enabling Real-Time Detection of Electrochemical Desorption Phenomena with Sub-Monolayer Sensitivity. *Electrochimica Acta* **2018**, *268*, 520–530. https://doi.org/10.1016/j.electacta.2018.02.060.
